# Perturbations in gut and respiratory microbiota in COVID-19 and influenza patients: a systematic review and meta-analysis

**DOI:** 10.3389/fmed.2024.1301312

**Published:** 2024-02-09

**Authors:** Xiu-Jie Chu, Dan-Dan Song, Ming-Hua Zhou, Xiu-Zhi Chen, Na Chu, Ming Li, Bao-Zhu Li, Song-Hui Liu, Sai Hou, Jia-Bing Wu, Lei Gong

**Affiliations:** ^1^Department of Acute Infectious Disease Prevention and Control, Anhui Provincial Center for Disease Control and Prevention, Hefei, Anhui, China; ^2^Department of Epidemiology and Biostatistics, School of Public Health, Anhui Medical University, Hefei, Anhui, China; ^3^School of Public Health, Bengbu Medical College, Bengbu, Anhui, China

**Keywords:** COVID-19, influenza, gut, respiratory tract, microbiome

## Abstract

**Objectives:**

Coronavirus disease-19 (COVID-19)/influenza poses unprecedented challenges to the global economy and healthcare services. Numerous studies have described alterations in the microbiome of COVID-19/influenza patients, but further investigation is needed to understand the relationship between the microbiome and these diseases. Herein, through systematic comparison between COVID-19 patients, long COVID-19 patients, influenza patients, no COVID-19/influenza controls and no COVID-19/influenza patients, we conducted a comprehensive review to describe the microbial change of respiratory tract/digestive tract in COVID-19/influenza patients.

**Methods:**

We systematically reviewed relevant literature by searching the PubMed, Embase, and Cochrane Library databases from inception to August 12, 2023. We conducted a comprehensive review to explore microbial alterations in patients with COVID-19/influenza. In addition, the data on α-diversity were summarized and analyzed by meta-analysis.

**Results:**

A total of 134 studies comparing COVID-19 patients with controls and 18 studies comparing influenza patients with controls were included. The Shannon indices of the gut and respiratory tract microbiome were slightly decreased in COVID-19/influenza patients compared to no COVID-19/influenza controls. Meanwhile, COVID-19 patients with more severe symptoms also exhibited a lower Shannon index versus COVID-19 patients with milder symptoms. The intestinal microbiome of COVID-19 patients was characterized by elevated opportunistic pathogens along with reduced short-chain fatty acid (SCFAs)-producing microbiota. Moreover, Enterobacteriaceae (including *Escherichia* and *Enterococcus*) and *Lactococcus*, were enriched in the gut and respiratory tract of COVID-19 patients. Conversely, Haemophilus and Neisseria showed reduced abundance in the respiratory tract of both COVID-19 and influenza patients.

**Conclusion:**

In this systematic review, we identified the microbiome in COVID-19/influenza patients in comparison with controls. The microbial changes in influenza and COVID-19 are partly similar.

## 1 Introduction

Coronavirus disease-19 (COVID-19), a global pandemic caused by the novel coronavirus severe acute respiratory syndrome coronavirus 2 (SARS-CoV-2), poses unprecedented challenges to both the world economy and healthcare services (1). Contrary to initial public optimism, COVID-19 appears to be accompanied by influenza for an extended period of time. COVID-19 patients generally manifest with fever, cough, dyspnea and gastrointestinal symptoms (2). Research has demonstrated that symptoms of the gastrointestinal tract may precede respiratory symptoms (3, 4). However, the underlying pathogenic mechanisms and factors influencing COVID-19 remain unclear. Furthermore, understanding the similarities and differences between influenza and COVID-19 is crucial as they may interact or coexist. Several factors have been identified to affect the severity and mortality rate of COVID-19 patients (5, 6), with the microbiome emerging as a significant environmental factor requiring urgent investigation.

The human microbiome represents a complex microecosystem that plays a vital role in maintaining health. Various microbes interact and grow together in healthy individuals, forming a strict symbiotic relationship (7, 8). Owing to the specificity of microbial niches, the composition and function of microorganisms vary according to different parts of the human body, such as the gastrointestinal tract, skin, and airway (9). Microorganisms are currently considered to be associated with the development and progression of various diseases.

While severe acute respiratory syndrome coronavirus 2 (SARS-CoV-2) primarily targets the respiratory tract. However, through several pathways characterized by enhanced intestinal epithelial-expressed angiotensin-converting enzyme 2 (ACE2) receptor, transmembrane protease serine 2 (TMPRSS2), and TMPRSS4, the gastrointestinal tract can also be infected with SARS-COV-2 (10, 11). Extensive research has demonstrated a dysbiotic microbiome in COVID-19 patients compared to no COVID-19 controls/influenza. Through 16S ribosomal RNA (rRNA) sequencing of the gut microbiome, Gu et al. found that COVID-19 patients were characterized by decreased α diversity compared to healthy controls, whereas showed increased Shannon index when compared with H1N1 patients (12). Moreover, metagenomic sequencing results revealed that several pharyngeal microbiomes were positively related to severe COVID-19 patients and elevated systemic inflammation markers, including Klebsiella spp. and Acinetobacter spp. (13). Meanwhile, COVID-19 patients, especially severe patients, exhibited a lower Shannon index compared with influenza B patients (13). A recent study reported a partially restored gut and throat swab microbiome of COVID-19 patients during hospitalization, although at a slower rate (14). Nevertheless, consistent robust findings have not been reported thus far. Moreover, the differences and similarities between influenza, a common respiratory infectious disease that currently breaks out frequently, and COVID-19 remain incompletely understood. Therefore, a systematic review of existing reports on the microbial change in COVID-19/influenza is critical.

## 2 Methods

### 2.1 Search strategy

On February 9, 2023, we retrieved the Embase, Cochrane Library, and PubMed databases to screen case–control studies on the microbiome of COVID-19/influenza patients. On August 12, 2023, we performed an update search on the database. The keywords included “Microbiome (MeSH)” or “Microbiota” or “Microbial Community” or “Microbial Community Composition” or “Flora” or “Microflora” AND “COVID-19 (Mesh)” or “Coronavirus Disease 2019” or “SARS-CoV-2” or “COVID-19 Pandemic.” “Microbiome (MeSH)” or “Microbiota” or “Microbial Community” or “Microbial Community Composition” or “Flora” or “Microflora” and “Influenza Human (MeSH)” or “Human Influenzas” or “Influenza” or “Influenzas” or “Human Flu” or “Flu, Human” or “Human Influenza” or “Influenza in Humans” or “Influenza in Human” or “Grippe.”

### 2.2 Selection criteria for research articles

The inclusion criteria were: (1) an observational study that investigated microbial differences in COVID-19/influenza patients compared with controls, and influenza-like illness patients were also included; (2) collected digestive tract samples (fecal and colonic tissue) or respiratory tract samples (lung, nasopharyngeal swabs, tongue coating and oropharyngeal swab samples); (3) human studies; and (4) English language. Studies were excluded if they (1) did not provide the required microbiome data; and (2) were conference abstracts, comments, or reviews.

### 2.3 Choice of outcome

First, the main outcome was the comparison of microbial species (operational taxonomic units (OTUs), other taxonomic entities) in COVID-19/influenza patients versus controls. Then, data on microbial diversity (α and β diversity) were collected. The primary statistical tables were classified into five types, according to previous studies. Including (1) studies collecting digestive tract samples or respiratory tract samples from COVID-19/influenza patients compared with no COVID-19/influenza controls. (2) studies collecting digestive tract samples or respiratory tract samples from recovered COVID-19 patients compared with no COVID-19 controls. (3) studies collecting digestive tract samples or respiratory tract samples from Long COVID-19 patients compared with no COVID-19 controls. (4) Studies comparing digestive tract samples or respiratory tract samples from COVID-19 patients compared with more severe symptoms versus controls (e.g., severe group versus mild group, COVID-19 groups versus recovered groups). (5) Studies comparing COVID-19 patients with other disease patients. Differences were considered statistically significant at *p* < 0.05.

Two researchers (X.J.C. and D.D.S.) independently conducted research selection based on initial screening of title and abstract, followed by a full-text review of eligible articles. Any disputes were resolved through consultation with a third researcher (L.G.). Two researchers (X.J.C. and M.H.Z.) independently extracted the data using a preset template.

A descriptive literature synthesis was performed to determine changes in microbial abundance. Data on α diversity (e.g., observed species, Shannon index, Simpson diversity index, inverse Simpson index, and Chao1 index) were collected for the meta-analysis. The standardized mean differences (SMDs) and 95% confidence interval between COVID-19/influenza patients and controls were calculated using an inverse-variance random-effects meta-analysis. DerSimonian-Laird estimator was used to quantify the inter-study heterogeneity, which was interpreted based on the *I*^2^ statistic. The median and 75% confidence intervals were collected to convert to mean and standard deviation, as previously described (15). When studies only provided statistical graphs, the required data was extracted from the graphs using WebPlot Digitizer V.4.42 (16). In cases where studies compared several subgroups (e.g., mild, moderate, severe, and decreased groups) with the same control, the number of control group was evenly into several groups, and the means and standard deviations remained unchanged, while the means and standard deviations remained unchanged. Effect size was classified as small (SMD = 0.2), moderate (SMD = 0.5), or large (SMD = 0.8). The heterogeneity among studies was calculated using the *I*^2^ statistic, with *I*^2^ > 50% was considered high heterogeneity. Meanwhile, other information including publication year, participant demographics and methodology was also extracted. Subgroup analyses and meta-regression based on several aspects (regional distribution, age group, and type of individuals) of the included participants were performed.

### 2.4 Quality assessment

The quality of the included studies was evaluated using the Newcastle-Ottawa Scale (NOS), a validated tool for assessing the quality of nonrandomized studies in systematic reviews.

For the changes in microbial abundance, we divided them into the following types according to certain rules. (1) Increased or decreased from one comparative group (at least three studies reported); and (2) inconsistent changes were defined as <75% comparative groups reported concordance with the findings.

## 3 Results

### 3.1 Study selection

**Figure 1:**
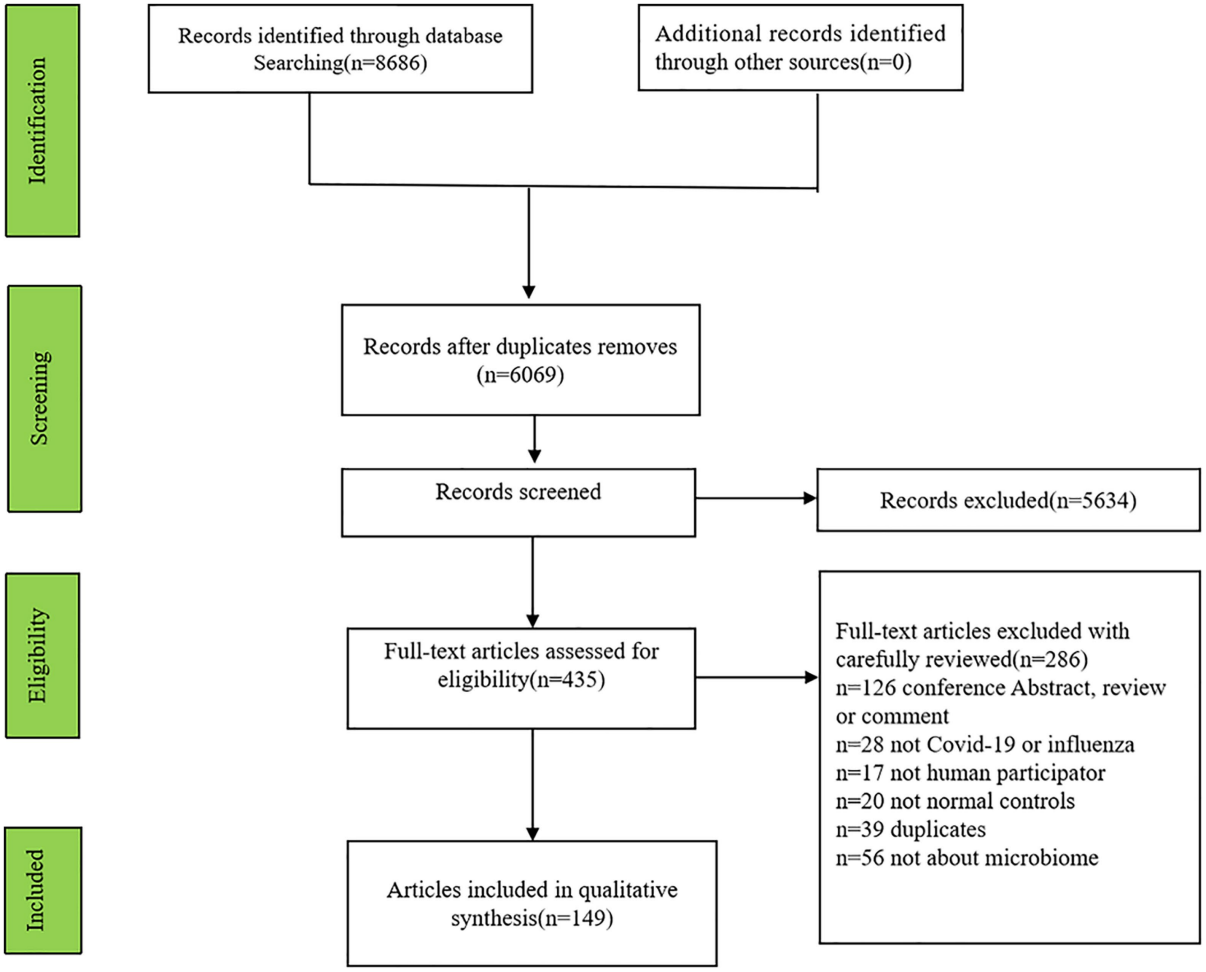
The flowchart of articles selection for inclusion in this systematic review.

Initially, 8,686 articles (2,919 articles involving influenza and 5,767 articles including COVID-19) were searched and 2,617 articles were excluded for repetition. Finally, 149 articles (131 involving COVID-19, 15 involving influenza, 3 articles involving COVID-19 and influenza patients) were identified (Figure 1).

### 3.2 Characteristics of included studies

The characteristics of the selected studies are shown in Supplementary Tables S1, S2. In total, this study encompassed 134 articles investigating COVID-19 along with 18 articles examining influenza. Among 134 articles involving COVID-19, 38 articles collecting gut samples and 57 articles collecting respiratory tract samples compared COVID-19 patients (*n* = 4,787) with no COVID-19 controls (*n* = 3,815). 13 articles collecting gut samples and five articles collecting respiratory tract samples compared recovered COVID-19 patients (*n* = 647) with no COVID-19 controls (*n* = 678). Three articles collecting gut samples and one article collecting respiratory tract samples compared long COVID-19 patients (*n* = 195) with no COVID-19 controls (*n* = 150). 22 articles collecting gut samples and 11 articles collecting respiratory tract samples compared COVID-19 patients with more severe symptoms (*n* = 1,425) versus controls (*n* = 1,544). Seven articles collecting gut samples and 13 articles collecting respiratory tract samples compared COVID-19 patients (*n* = 828) with no COVID-19 patients (*n* = 609). Among 18 articles involving COVID-19, four articles collecting gut samples and 14 articles collecting respiratory tract samples compared influenza patients (*n* = 1,617) with no influenza controls (*n* = 1,036).

### 3.3 α diversity

#### 3.3.1 α diversity in studies collecting gut samples

The α diversity serves as a comprehensive indicator that reflects the richness and evenness of community structure, encompassing key indices such as the Shannon index, Simpson index, Chao1 index, and ACE.

**Figure 2:**
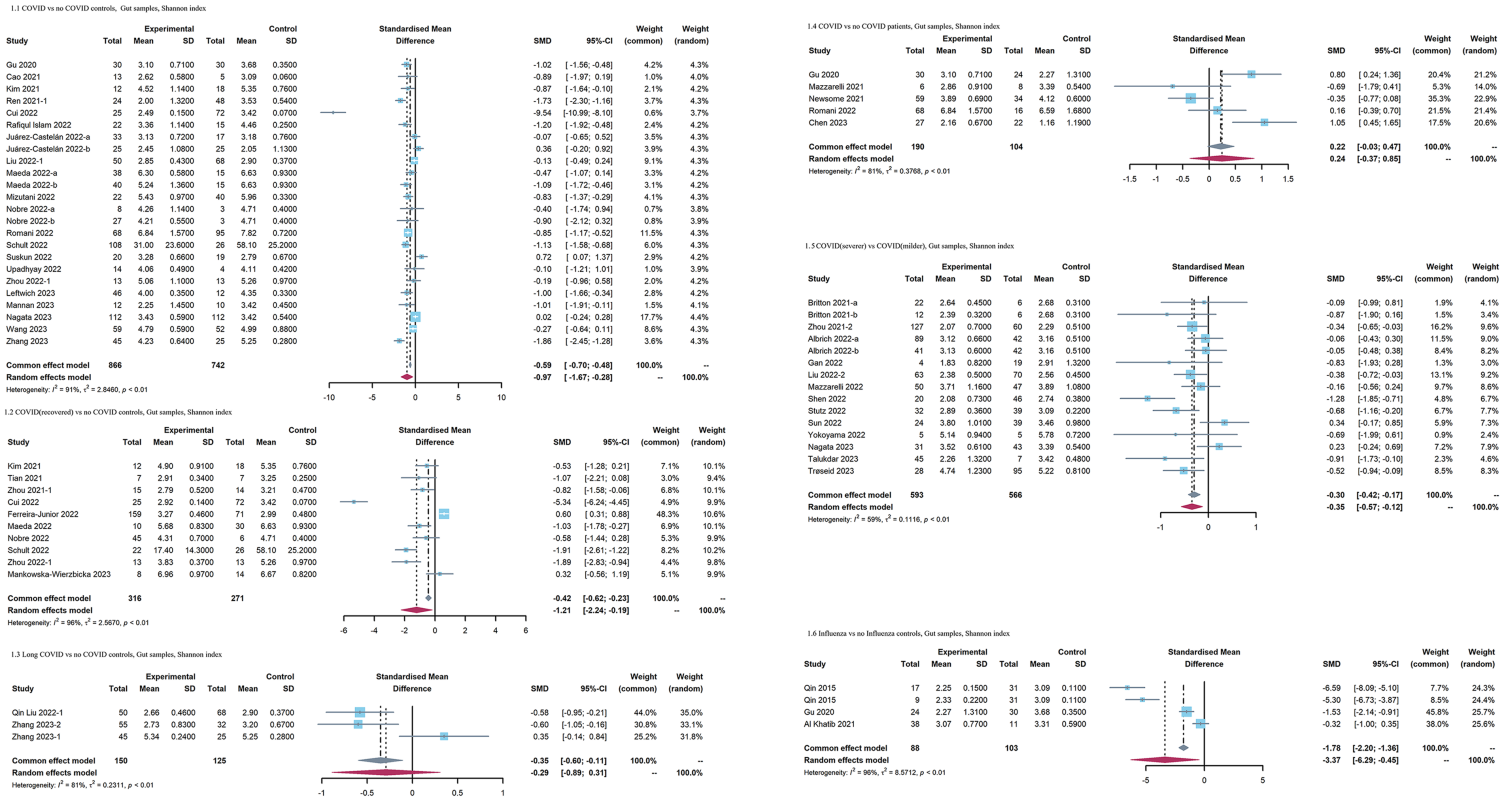
Forest plots of Shannon index in the gut microbiota of patients with COVID-19/influenza patients. SD, standard deviation; CI, confidence interval.

When COVID-19 patients were compared with no COVID-19 controls, the pooled estimate indicated that the Shannon index of the intestinal microbiota was significantly reduced in patients with COVID-19 (SMD = −0.97 [95% CI = −1.67, −0.28], *p* < 0.01, inverse-variance, random-effect, *I*^2^ = 91%) (Figure 2). However, no significant difference was observed in the Simpson diversity index for the COVID-19 group [SMD = −1.33 [95% CI = −3.37, 0.71], *p* < 0.01, inverse-variance, random-effect, *I*^2^ = 97% (Supplementary Figure S1)]. In terms of richness, no significant differences in the observed species (SMD = −1.63 [95%CI = −4.12, 0.87], *p* < 0.01, inverse-variance, random-effect, *I*^2^ = 96%) and Chao1 index (SMD = −1.92 [95%CI = −4.93, 1.08], *p* < 0.01, inverse-variance, random-effect, *I*^2^ = 97%) were observed in the COVID-19 patients (Supplementary Figure S1). When recovered COVID-19 patients were compared with no COVID-19 controls, the Shannon index was significantly decreased in patients with recovered COVID-19 (SMD = −1.21 [95% CI = −2.24, −0.19], *p* < 0.01, inverse-variance, random-effect, *I*^2^ = 96%). Furthermore, when comparing COVID-l9 patients with more severe symptoms to controls, patients with more severe COVID-19 exhibited a slightly lower effect size of Shannon index [SMD = −0.35 [95%CI = −0.57, −0.12], *p* < 0.01, inverse-variance, random-effect, *I*^2^ = 59% (Figure 2)]. When COVID-19 patients were compared with no COVID-19 patients, mainly including patients with other respiratory symptoms, such as flu or pneumonia, the Shannon index showed no significant difference (SMD = 0.24 [95%CI = −0.37, 0.85], *p* < 0.01, *I*^2^ = 81%, inverse-variance, random-effect). Regarding influenza, only three studies provided data on the Shannon index. Influenza patients had a lower effect size with high heterogeneity [SMD = −3.37 [95%CI = −6.29, −0.45], *p* < 0.01, inverse-variance, random-effect, *I*^2^ = 96% (Figure 2)].

#### 3.3.2 α diversity in studies collecting respiratory tract samples

**Figure 3:**
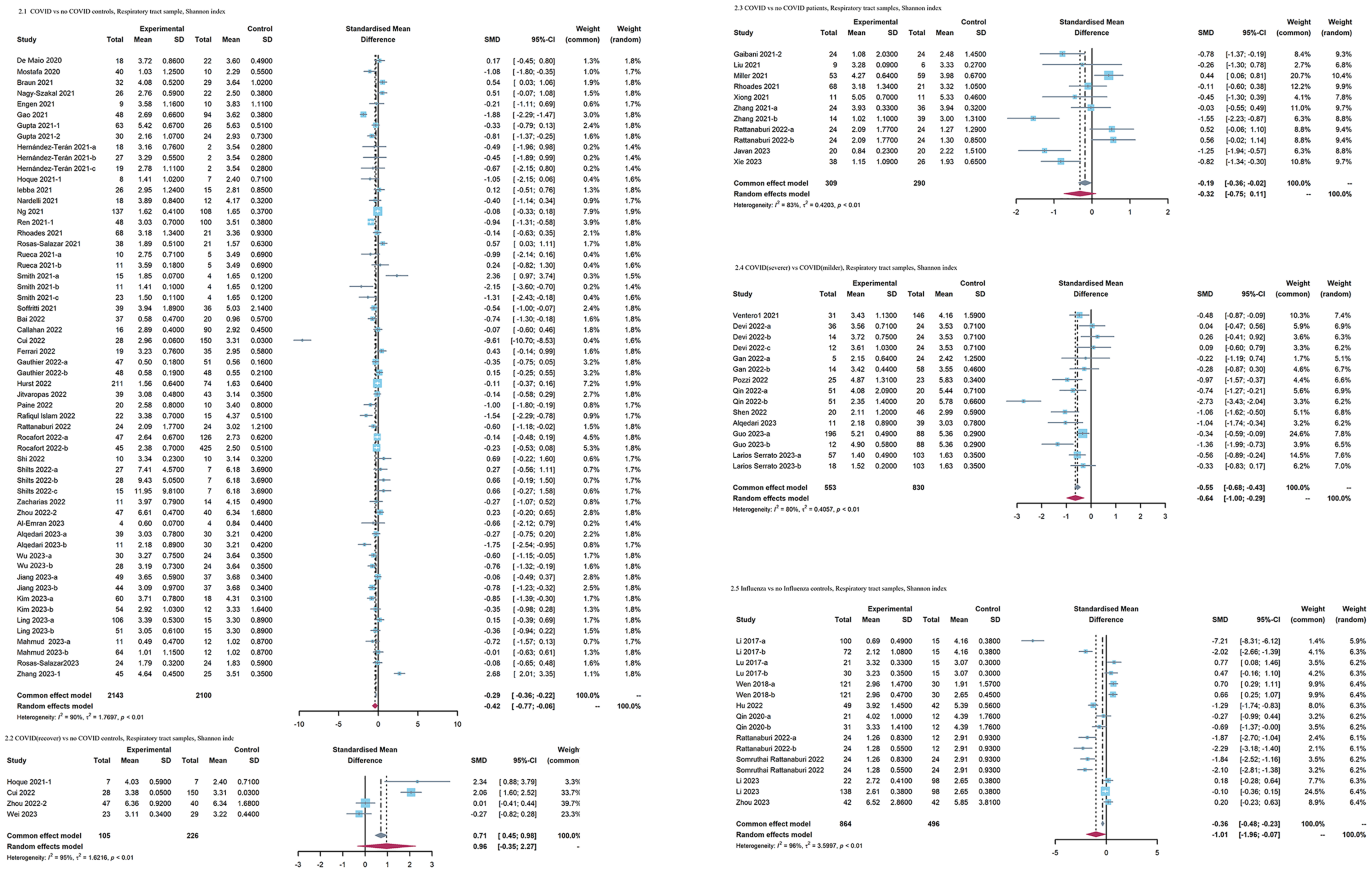
Forest plots of Shannon index in the respiratory microbiota of patients with COVID-19/influenza patients. SD, standard deviation; CI, confidence interval.

When COVID-19 patients were compared to no COVID-19 controls, as for index reflecting evenness, an overall small effect size of the Shannon index was also observed in COVID-19 patients [SMD = −0.42 [95%CI = −0.77, −0.06], *p* < 0.01, *I*^2^ = 90%, inverse-variance, random-effect (Figure 3)]. No significant differences in the Simpson diversity index (SMD = −0.65 [95%CI = −1.41, 0.11], *p* < 0.01, *I*^2^ = 94%, inverse-variance, random-effect) and Simpson’s reciprocal index (SMD = 0.05 [95%CI = −0.13, 0.23], *p* = 0.54, *I*^2^ = 0%, inverse-variance, random-effect) were observed in the COVID-19 patients (Supplementary Figure S1). As for richness, the observed species (SMD = −0.93 [95%CI = −0.73, −0.12], *p* < 0.01, *I*^2^ = 95%, inverse-variance, random-effect) was decreased, whereas Chao1 (SMD = −1.01 [95%CI = −2.45, 0.43], *p* < 0.01, *I*^2^ = 96%, inverse-variance, random-effect) showed no significant difference in COVID-19 patients (Supplementary Figure S1). the Shannon index in recovered COVID-19 patients had no significant change versus no COVID-19 controls (SMD = 0.96 [95% CI = −0.35, 2.27], *p* < 0.01, inverse-variance, random-effect, *I*^2^ = 95%). COVID-19 patients with more severe symptoms had a lower effect size on the Shannon index than COVID-19 patients with milder symptoms [SMD = −0.64[95%CI = −1, −0.29], *p* < 0.01, *I*^2^ = 80%, inverse-variance, random-effect (Figure 3)]. When COVID-19 patients were compared with no COVID-19 patients, the Shannon index showed no significant difference (SMD = −0.32 [95%CI = −0.75, 0.11], *p* < 0.01, *I*^2^ = 83%, inverse-variance, random-effect). When comparing influenza patients with no influenza control, the influenza patients exhibited lower Shannon index (SMD = −1.01 [95%CI = −1.96, −0.07], *p* < 0.01, *I*^2^ = 96%, inverse-variance, random-effect).

#### 3.3.3 Subgroup analysis and sensitivity analysis

To further explore the source of inter-study heterogeneity affecting the gut and respiratory microbiome of COVID-19 patients, we performed further subgroup analyses and meta-regression based on several aspects (regional distribution, age group, and type of individuals) of the included participants. However, the subgroup analyses and meta regression seem not to explain the source of the mutation (Supplementary Figures S2, S3; Supplementary Tables S3, S4). Sensitivity analysis was performed by sequentially excluding individual studies to assess both heterogeneity sources and result stability. Nevertheless, the result did not change substantially (*I*^2^ ranged from 81.3 to 90.4 in the respiratory tract and 80.7 to 91.6 in the gut).

### 3.4 β diversity

In studies collecting gut samples, the vast majority of studies reported altered microbial β diversity in the case group versus the control group (16/17 articles for the comparison group of COVID-19 vs. no COVID-19 controls, 5/6 articles for the comparison group of recovered COVID-19 vs. no COVID-19 controls, 10/13 articles for the comparison group of COVID-19 with severer symptoms vs. COVID-19 with milder symptoms, 5/5 articles for the comparison group of COVID-19 vs. no COVID-19 patients, 3/3 articles for the comparison group of influenza vs. no influenza controls).

Similarly, in studies collecting respiratory tract samples, most studies reported altered microbial β diversity in the case group versus the control group (25/34 articles for the comparison group of COVID-19 vs. no COVID-19 controls, 3/4 articles for the comparison group of recovered COVID-19 vs. no COVID-19 controls, 8/9 articles for the comparison group of COVID-19 with severer symptoms vs. COVID-19 with milder symptoms, 8/8 articles for the comparison group of COVID-19 vs. no COVID-19 patients, 9/9 articles for the comparison group of influenza vs. no influenza controls).

### 3.5 The abundance differences of microbiome

#### 3.5.1 The abundance differences of microbiome in COVID-19 patients comparing with controls

**Figure 4:**
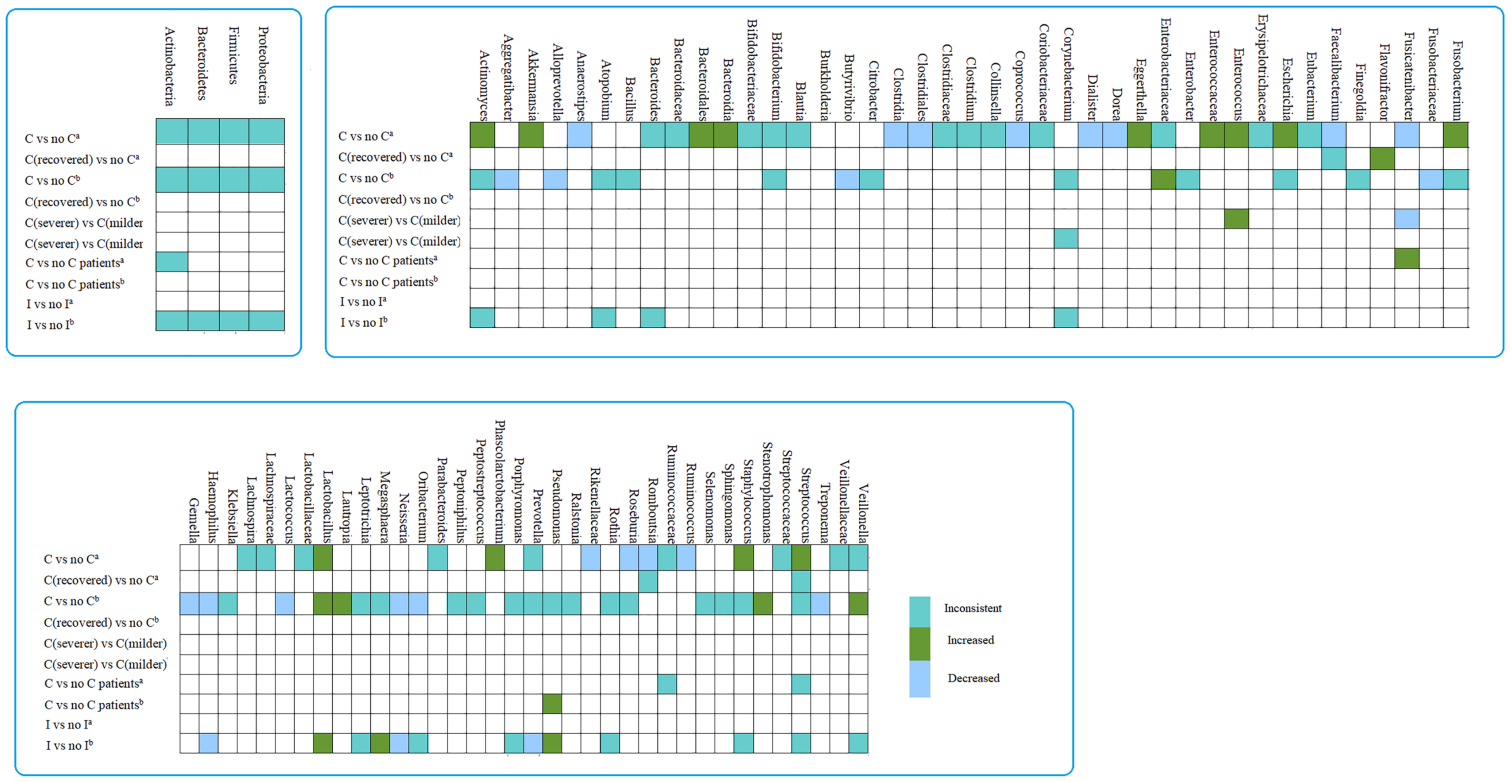
The differences in abundance taxa of microbiome in COVID-19/influenza versus control. a, gut sample; b, Respiratory tract sample; I, influenza patients.

In studies collecting gut samples and comparing COVID-19 patients with no COVID-19 controls, Bacteroidia (class), Bacteroidales (order), *Actinomyces*, *Akkermansia*, *Eggerthella*, *Enterococcus*, *Escherichia*, *Fusobacterium*, *Lactobacillus*, *Phascolarctobacterium*, *Staphylococcus*, *Streptococcus* were considered enriched in COVID-19 patients as established rules. Clostridia (class), Clostridiales (order), Rikenellaceae (family), *Anaerostipes*, *Coprococcus*, *Dialister*, *Dorea*, *Faecalibacterium*, *Fusicatenibacter*, *Roseburia*, *Romboutsia* and *Ruminococcus* were less abundant in patients with COVID-19. *Flavonifractor* was increased in recovered COVID-19 patients. In studies comparing COVID-19 patients with more severe symptoms versus controls, *Enterococcus* was enriched in COVID-19 patients with more severe symptoms, whereas *Fusicatenibacter* were less abundant in COVID-19 patients with more severe symptoms (Figure 4; Supplementary Tables S5, S7).

In studies collecting respiratory tract sample, when comparing COVID-19 patients versus no COVID-19 controls, Enterobacteriaceae (family), *Lactobacillus*, *Lautropia*, *Stenotrophomonas* and *Veillonella* was enriched in COVID-19 patients, Fusobacteriaceae (family), *Aggregatibacter*, *Alloprevotella*, *Butyrivibrio*, *Gemella*, *Haemophilus*, *Lactococcus, Neisseria, Oribacterium* and *Treponema* were decreased in COVID-19 patients. Moreover, *Lactobacillus*, *Megasphaera*, and *Pseudomonas* were considered to be increased in influenza patients. *Haemophilus*, *Neisseria*, *Prevotella* were less abundant in patients with influenza (Figure 4 and Supplementary Tables S5, S7).

## 4 Discussion

This study focused on investigating the gut and respiratory tract microbiome of patients with COVID-19 and influenza. Additionally, a comprehensive analysis was conducted to explore the variations in microbiome among COVID-19 patients with different disease severities. The key findings of this study were as follows: (1) The Shannon index was more likely to decrease in patients with COVID-19/influenza versus no COVID-19/influenza controls. Meanwhile, The Shannon index of the gut and respiratory tract samples was also more likely to decrease in COVID-19 patients with more severe symptoms. (2) Most studies reporting β-diversity showed altered β-diversity in COVID-19/influenza patients. (3) In terms of specific microbial abundance changes, the intestinal microbiome of COVID-19 patients was characterized by elevated opportunistic pathogens along with reduced short-chain fatty acid (SCFAs)-producing microbiota. Similarly, both COVID-19 and influenza patients showed increased levels of Lactobacillus while experiencing reduced levels of Haemophilus and Neisseria within their respiratory tracts.

In this meta-analysis, the Shannon index of COVID-19/influenza patients was reduced versus no COVID-19/influenza controls, which is consistent with the results of previous researches (17, 18). Furthermore, when comparing COVID-19 patients with non-COVID-19 patients primarily presenting other respiratory symptoms such as flu and pneumonia, the Shannon index showed no significant difference. Meanwhile, the microbial abundance changes in COVID-19 and influenza were partially similar. The above evidence indicates that the microbial structure of COVID-19 is more likely to be similar to influenza. Several articles have described the microbial structures in COVID-19 patients compared to influenza patients, however, there were no consistent changes in these studies. Rattanaburi et al. employed 16S sequencing to conduct a comparative analysis of the upper respiratory tract microbiota in patients afflicted with influenza and COVID-19, the Shannon diversity index in influenza A and B groups exhibited a significantly lower level compared to that observed in non-influenza and COVID-19 groups (19). Opposite findings were obtained from another study employing metagenomic sequencing, the species-level alpha-diversity of the oropharyngeal microbiota is significantly reduced in COVID-19 patients compared to influenza patients, particularly among critically ill individuals with COVID-19 (13). Notably, both diseases share similarities including targeting respiratory epithelium cells and exhibiting similar symptoms (20, 21). Given their global impact as mainstream respiratory diseases affecting billions of people worldwide (22), it is crucial to enhance our understanding of differential diagnosis and distinctions between COVID-19 and influenza. However, the current understanding of SARS-CoV-2 and influenza remains inadequate. In the future, researchers need to further explore the underlying mechanisms. For example, whether consider microbial regulation methods such as fecal transplantation to treat influenza, COVID-19 and the long-term symptoms they cause in the future treatment strategy.

In this study, the respiratory and intestinal microbiomes of COVID-19 patients displayed distinct changes in microbial abundance. The intestinal microbiome is characterized by rising opportunistic pathogens (*Enterococcus*, *Escherichia*, *Eggerthella*, *Fusobacterium*) (23–25) and relatively diminished short-chain fatty acid (SCFAs) -producing microbiota (*Anaerostipes*, *Coprococcus*, *Faecalibacterium*, *Roseburia*) (26, 27). Interestingly, three studies reported increased *Flavonifractor* in recovered COVID-19 patients versus no COVID-19 controls. *Flavonifractor* is generally considered a butyrate-producing microbiota (27), suggesting potential improvement in the gut microbiome during recovery from COVID-19. *Pseudomonas*, as an opportunistic pathogen with drug resistance (28), was found to be enriched in the respiratory tract of patients with influenza.

It is worth noting that Enterobacteriaceae (including *Escherichia* and *Enterococcus*), as opportunistic pathogens, were not only enriched in the gut and respiratory tract of COVID-19 patients, but also in the gut of severe COVID-19 patients. *Enterococcus* is one of the most important pathogens causing healthcare associated infections (29). Recent research has revealed the gene expression of platelet aggregation and neutrophil degranulation was positively related to *Enterococcus faecalis* (30), especially in patients with severe COVID-19, suggesting potential involvement of the microbiome in COVID-19 progression. Furthermore, *Enterococcus* exhibits resistance to several commonly used antimicrobials (31), which may impede the recovery process of COVID-19 patients. Several SCFAs-producing microbiota were reduced in the gut of COVID-19 patients. Meanwhile, research has demonstrated that even after recovery from COVID-19, the depletion of SCFA-producing microbiota remains sustained (32). Depletion of SCFA-producing microbiota has been associated with various diseases including inflammatory bowel disease, depression, and rheumatoid arthritis (33–35). SCFAs are microbial metabolites with anti-inflammatory effects. The infection of SARS-CoV-2 may lead to disturbances in the intestinal environment, and eventually destroy the living environment of other symbiotic bacteria (27). *Haemophilus* is a common pathogen in human (36), although several studies have reported reduced *Haemophilus* in the respiratory tract of patients with COVID-19 and influenza. However, *Haemophilus influenzae*, a resident microbiome within the upper respiratory tract capable of causing secondary respiratory infections, is more likely to be increased in the respiratory tract of COVID-19 patients (37). The future necessitates further investigation into the underlying factors of changes in *Haemophilus* by researchers.

The upper respiratory tract and lower respiratory tract demonstrated similar microbial structures; one hypothesis is that the pathogenic microorganisms in oropharyngeal secretions may “micro aspirate” into the lungs (38, 39). Disturbances in the balance between migration and elimination of the lung microbiome during lung disease can lead to alterations in its composition, with bacteria possessing a competitive advantage becoming dominant. The epithelial barrier of the gut prevents the invasion of pathogenic microorganisms and helps maintain tolerance to food antigens; it may also be involved in systemic and pulmonary immune functions. Once compromised, microbes can migrate to the bloodstream or lungs, triggering sepsis or acute respiratory distress syndrome (40, 41). Meanwhile, some metabolites of gut microbiota in digestion, including SCFAs, can affect the human immune system, which in turn can control inflammation in the lungs. Accumulating evidence underscores the interplay and connection between the gut and lungs known as the gut-lung axis (42–44).

The composition and functional homeostasis of the human microbiota play a crucial role in maintaining and regulating normal immune function as well as combating infections (45, 46). The microbiome between the respiratory tract and gut exists in complex interactions. First, SARS-CoV-2 infection can induce lung tissue damage and trigger cytokine storms by promoting pro-inflammatory pathways such as NF-κB and TNF pathways (47–49). On the other hand, intestinal infection can lead to direct damage to the intestinal structure, further causing inflammation, and eventually leading to the disorder of gut and respiratory microbiota (50). In the gut and respiratory tract of COVID-19 patients, the absence of beneficial bacteria or the overgrowth of opportunistic pathogens may exacerbate the inflammatory response in the body, leading to the exacerbation of symptoms.

This study has several limitations. First, the lack of data on microbial diversity and abundance changes in numerous studies hinders the acquisition of robust conclusions. Additionally, there is currently no consistent standard for defining severe and mild COVID-19 patients in each articles. However, considering the necessity of investigating the microbial differences in COVID-19 patients with different severity, we extracted microbiome data from people with more severe symptoms (for example, Severe group, ICU group, and deceased group) versus patients with milder symptoms (for example., mild group, no ICU group, and live group). In future research endeavors, it is imperative to explore microbial changes across different levels of COVID-19 severity. Long-term cohorts are also in urgent need of investigating microbial changes and potential influencing mechanisms during recovery, particularly given the growing attention to the issue of ‘long COVID-19.’ Moreover, the meta-analysis exhibits a high degree of heterogeneity, which may impact the robustness of the conclusions. High heterogeneity is a common problem in current microbiome-related meta-analyses, not only in COVID-19, but also in other diseases (16). The elevated heterogeneity could be attributed to various factors such as variations in sequencing methods employed across studies and unavailability of raw data within any included studies. Despite employing multiple approaches to explore the sources of heterogeneity, satisfactory results were not obtained. Finally, we are not clear about the potential impact of drugs (antibiotics and proton pump inhibitors) and other demographic factors on the microbiome throughout the entire process of onset and recovery.

## 5 Conclusion

In this comprehensive review and meta-analysis, our results demonstrated the microbial composition of COVID-19 patients may be similar with influenza patients, manifested as declining Shannon index and similar microbial abundance change. After clearing the disease state, some SCFA-producing microbiota may be partially restored after clearance of SARS-CoV-2. Moreover, COVID-19 with different severity exhibits distinct microbial construction.

## Data availability statement

The original contributions presented in the study are included in the article/Supplementary material, further inquiries can be directed to the corresponding author.

## Author contributions

X-JC: Data curation, Methodology, Writing – original draft. D-DS: Data curation, Methodology, Writing – original draft. M-HZ: Data curation, Methodology, Writing – original draft. X-ZC: Formal analysis, Validation, Writing – original draft. NC: Formal analysis, Data curation, Writing – original draft. ML: Formal analysis, Project administration, Writing – original draft. B-ZL: Conceptualization, Investigation, Writing – review & editing. S-HL: Software, Writing – original draft. SH: Software, Writing – review & editing. J-BW: Formal analysis, Writing – review & editing. LG: Investigation, Writing – review & editing.
