# Supplementary material for: Perturbations in gut and respiratory microbiota in COVID-19 and influenza patients: a systematic review and meta-analysis
Source: Front Med (Lausanne). 2024 Feb 9;11:1301312. doi: 10.3389/fmed.2024.1301312 (PMC10884097; doi:10.3389/fmed.2024.1301312)
Supplement: Supplementary file 1 [file Data_Sheet_1.docx]

Supplementary table 1 Characteristic of included articles involving COVID-19

| First author & publication year | Country | Specimen | Disease | Control | Numbel (D/C) | Age (D/C) | Male % ( D/C) | Sequencing Platform | Preservation condition | Microbiome assessment method | Multiple comparisons correction? |
| --- | --- | --- | --- | --- | --- | --- | --- | --- | --- | --- | --- |
|  |  |  |  |  |  |  |  |  |  |  |  |
| Covid-19 vs no Covid-19 control Gut | | | | |  |  |  |  |  |  |  |
| Gu 2020^1^ | China | Stool | COVID | HC | 30/30 | 55/53.5 | 43.3/43.3 | Illumina Platform | -80°C | 16S rRNA gene sequencing ( V3-V5) | Yes |
| Zuo 2020^2^ | China | Stool | COVID( antibiotics-naïve) | HC | 7/15 | NR/48 | NR/60 | Illumina Platform | NR | Shotgun metagenomic sequencing | NR |
| Bataineh 2021^3^ | United Arab Emirates | Stool | COVID | HC | 86/57 | NR/NR | 60.5/24.6 | Illumina Platform | -80°C | 16S rRNA gene sequencing ( V3,V4) | NR |
| Cao 2021^4^ | China | Stool | COVID | HC | 13/5 | 48/NR | 46.2/NR | Illumina Platform | NR | Metagenomic sequencing analyses | Yes |
| Gaibani 2021-1^5^ | Italy | Stool | COVID | HC | 69/69 | 73/59 | 55.1/43.5 | Illumina Platform | -80°C | 16S rRNA gene sequencing( V3-V4) | Yes |
| He 2021^6^ | China | Stool | COVID | HC | 13/21 | 26.9/43 | 76.9/57.1 | NR | -80°C | Metaproteomics | Yes |
| Kim 2021^7^ | Korea | Stool | COVID | HC | 12/36 | 26/NR | 66.7/NR | Illumina Platform | NR | 16S rRNA gene sequencing ( V3,V4) | Yes |
| Khan 2021^8^ | India | Stool | COVID(asymptomatic) | HC | NR/10 | 57.9/NR | NR/NR | NR | NR | 16S rRNA gene sequencing ( V3–V4) | NR |
|  |  |  | COVID(Mild) | HC | NR/10 | 49.4/NR | NR/NR |  |  |  |  |
|  |  |  | COVID(Severe) | HC | NR/10 | 45.9/NR | NR/NR |  |  |  |  |
| Li 2021^9^ | China | Stool | COVID | HC | 37/10 | 44.1/37.4 | 51.4/70 | Bgiseq-500 Platform | -80°C | Metagenome Sequencing | Yes |
| Ren 2021-1^10^ | China | Stool | COVID | HC | 24/48 | 48./48.5 | 58.3/52.1 | Illumina Platform | -80°C | 16S rRNA gene sequencing (V3-V5) | NR |
| Wu 2021^11^ | China | Stool | COVID | HC | 50/32 | 45/46.5 | 63.9/65.6 | Illumina Platform | NR | 16S rRNA gene sequencing (V3-V4) | Yes |
| Xu 2021^12^ | China | Stool | COVID | HC | 9/14 | 6.4/5.6 | 44.4/67.5 | Illumina Platform | -80°C | 16S rRNA gene sequencing ( V4) | Yes |
| Yeoh 2021^13^ | China | Stool | COVID | No COVID | 87/78 | 35.6/45.5 | 54/42.3 | Illumina Platform | -80°C | Shotgun metagenomic sequencing analyses | NR |
| Cui 2022^14^ | China | Stool | COVID | HC | 25/72 | 47.2/47 | 32/34.7 | Illumina Platform | -80°C | 16S rRNA gene sequencing ( V3-V5) | NR |
| Cuenca 2022^15^ | Spain | Stool、Rectal Swab | COVID(ICU) | HC | 46/17 | 61/55 | 70/47.7 | Illumina Platform | -80°C | 16S rRNA gene sequencing ( V4) | Yes |
| Hazan 2022^16^ | America | Stool | COVID | No COVID | 50/20 | 53/48 | 48/35 | Illumina Platform | -20°C | Metagenome Sequencing | Yes |
| Juárez-Castelán 2022^17^ | Mexico | Rectal Swabs | COVID | No COVID | 33/17 | 25.5/25.5 | 0/0 | NR | -80°C | 16S rRNA gene sequencing (V3) | NR |
|  |  |  | COVID | No COVID | 25/25 | 0/0 | 68/68 |  |  |  |  |
| Liu 2022-1^18^ | China | Stool | COVID | No COVID | 50/68 | NR/47.2 | NR/47.1 | Illumina Platform | -80°C | Metagenome Sequencing | Yes |
| Maeda 2022^19^ | Japan | Stool | COVID(Mild) | HC | 38/30 | 72/64 | 57.9/66.7 | Illumina Platform | -20°C | 16S rRNA gene sequencing ( V1–V2)， | NR |
|  |  |  | COVID(Severe) | HC | 40/30 | 72/64 | 75/66.7 |  |  |  |  |
| Mizutani 2022^20^ | Japan | Stool | COVID | HC | 22/40 | 42/42 | 81.8/100 | Illumina Platform | -80°C | 16S rRNA gene sequencing ( V3–V4) | NR |
| Nobre 2022^21^ | Portugal | Stool | COVID(Mild) | HC | 8/7 | 64.5/33.4 | 62.5/42.9 | Illumina Platform | -20°C | 16S rRNA gene sequencing ( V3-V4) | NR |
|  |  |  | COVID(Severe) | HC | 27/7 | 63.6/33.4 | 66.7/42.9 |  |  |  |  |
| Rafiqul Islam 2022^22^ | Bangladesh | Stool | COVID | HC | 22/15 | NR/NR | NR/NR | Illumina Platform | NR | 16S rRNA gene sequencing ( V3–V4) | Yes |
| Romani 2022^23^ | Italy | Stool | COVID | HC | 68/95 | 6.5/NR | 56/NR | Illumina Platform | -80°C | 16S rRNA gene sequencing (V3-V4) | Yes |
| Schult 2022^24^ | Germany | Stool | COVID | No COVID | 108/26 | 62/63 | 54.6/57.7 | Illumina Platform | NR | 16S rRNA gene sequencing ( V3–V4) | Yes |
| Suskun 2022^25^ | Turkey | Stool | COVID | HC | 20/19 | 9.5/8 | 50/57.9 | Illumina Platform | -80°C | 16S rRNA gene sequencing ( V3–V4) | Yes |
| Sun 2022^26^ | China | Stool | COVID | No COVID | 63/8 | 48/37 | 61.9/37.5 | Illumina Platform | -80°C | Metagenome Sequencing | Yes |
| Upadhyay 2022^27^ | America | Stool | COVID | No COVID | 14/4 | 44.8/44.8 | 21.4/75 | Illumina Platform | -80°C | 16S rRNA gene sequencing (V4), Metagenome Sequencing | Yes |
| Xu 2022^28^ | China | Stool | COVID(Mild or Moderate) | HC | 24/31 | 51/NR | 70.8/NR | Illumina Platform | -80°C | Metagenome Sequencing | NR |
|  |  |  | COVID(Severe/Critical) | HC | 14/31 | 60.5/NR | 71.4/NR |  |  |  |  |
| Yin 2022^29^ | America | Stool | COVID | HC | 20/20 | 58/41 | 60/60 | Illumina Platform | -80°C | 16S rRNA gene sequencing ( V5) | Yes |
| Zhang 2022^30^ | China | Stool | COVID | No COVID | 66/70 | 42.6/45.8 | 59.1/41 | Illumina Platform | -80°C | Metagenome Sequencing | Yes |
| Zhou 2022-1^31^ | China | Stool | COVID | HC | 13/13 | 24/26 | 76/76 | Illumina Platform | -80°C | Metatranscriptome sequencing | Yes |
| de Nies 2023^32^ | Luxembourg | Stool | COVID | No COVID | 61/57 | 43.9/42.1 | 63.9/61.4 | Illumina Platform | -80°C | Metagenomic and metatranscriptomic sequencing | Yes |
| Gutiérrez-Díaz 2023^33^ | Spain | Stool | COVID | No COVID | 11/9 | 1.9/1.7(month) | 27.3/88,9 | Illumina Platform | -80°C | 16S rRNA gene sequencing (V3) | NR |
|  |  |  | COVID | No COVID | 8/9 | 17/11(month) | 37.5/55.6 |  |  |  |  |
| Leftwich 2023^34^ | America | Stool | COVID | HC | 46/12 | NR/NR | 0/0 | Illumina Platform | NR | 16S rRNA gene sequencing (V3-V4) | NR |
| Mannan 2023^35^ | Bangladesh | Stool | COVID | HC | 12/10 | 40.5/49.3 | 66.7/80 | Illumina Platform | -80°C | 16S rRNA gene sequencing (V3-V4) | Yes |
| Nagata 2023^36^ | Japan | Stool | COVID | HC | 112/112 | 33.9/33 | 65.2/63.4 | Illumina Platform | -80°C | Metagenome Sequencing | Yes |
| Wang 2023^37^ | China | Stool | COVID | HC | 59/52 | 4.8/5 | 49.2/30.8 | Illumina Platform | -80°C | 16S rRNA gene sequencing (V3-V4) | NR |
| Zhang 2023-1^38^ | China | Stool | COVID | HC | 45/25 | 40.5/35.6 | 60/52 | Illumina Platform | -80°C | Metagenome sequenc_x005f ing | Yes |
| Covid-19(recovered) vs no Covid-19 control Gut | | | | |  |  |  |  |  |  |  |
| Kim 2021^7^ | Korea | Stool | COVID(Recovered) | HC | 12/36 | 26/NR | 66.7/NR | Illumina Platform | NR | 16S rRNA gene sequencing ( V3,V4) | Yes |
| Tian 2021^39^ | China | Stool | COVID(Recovered) | HC | 7/7 | 38/32.8 | 100/100 | Illumina Platform | -80°C | 16S rRNA gene sequencing ( V3-V4) | NR |
| Yeoh 2021^13^ | China | Stool | COVID(Recovered) | No COVID | 100/78 | 36.4/45.5 | 53/42.3 | Illumina Platform | -80°C | Shotgun metagenomic sequencing analyses | NR |
| Zhou 2021-1^40^ | China | Stool | COVID(Recovered) | HC | 15/14 | 29/37.5 | 20/35.7 | Illumina Platform | -80°C | 16S rRNA gene sequencing ( V3–V4) | NR |
| Cui 2022^14^ | China | Stool | COVID(Recovered) | HC | 25/72 | 48.2/47 | 32/34.7 | Illumina Platform | -80°C | 16S rRNA gene sequencing ( V3-V5) | NR |
| Ferreira-Junior 2022^41^ | Brazil | Stool | COVID(Recovered) | HC | 159/71 | 42.5/46.1 | 32.1/28.2 | Illumina Platform | -80°C | 16S rRNA gene sequencing (V3,V4) | Yes |
| Maeda 2022^19^ | Japan | Stool | COVID(Recovered) | HC | 10/30 | NR/NR | NR/NR | Illumina Platform | -20°C | 16S rRNA gene sequencing ( V1–V2)， | NR |
| Nobre 2022^21^ | Portugal | Stool | COVID(Recovered) | HC | 45/7 | 56.3/33.4 | 22.2/42.9 | Illumina Platform | -20°C | 16S rRNA gene sequencing ( V3-V4) | NR |
| POLO 2022^42^ | Turkey | Stool | COVID(Recovered) | No COVID | 8/8 | 41/32 | 50/25 | Illumina Platform | -80°C | 16S rRNA gene sequencing ( V3-V4)，QPCR | NR |
| Schult 2022^24^ | Germany | Stool | COVID(Recovered) | No COVID | 22/26 | 65/63 | 81.8/57.7 | Illumina Platform | NR | 16S rRNA gene sequencing ( V3–V4) | Yes |
| Zhou 2022-2^43^ | China | Stool | COVID(Recovered) | HC | 13/13 | 24/26 | 76/76 | Illumina Platform | -80°C | Metatranscriptome sequencing | Yes |
| Mankowska-Wierzbicka 2023^44^ | Poland | Stool | COVID(Recovered) | HC | 8/14 | 34.9/37 | 37.5/78.6 | Illumina Platform | -80°C | 16S rRNA gene sequencing (V1-V9) | Yes |
| Zhang 2023-2^45^ | China | Stool | COVID(Recovered) | HC | 75/32 | 62/57.5 | 58.7/40.6 | Illumina Platform | -80°C | 16S rRNA gene sequencing (V3-V4) | NR |
| Long Covid-19 vs no Covid-19 control Gut | | | | |  |  |  |  |  |  |  |
| Liu 2022-1^18^ | China | Stool | COVID(long COVID-19) | No COVID | 50/68 | NR/47.2 | NR/47.1 | Illumina Platform | -80°C | Metagenome Sequencing | Yes |
| Zhang 2023-2^45^ | China | Stool | COVID(long COVID-19 ) | HC | 55/32 | 57/57.5 | 41.8/40.6 | Illumina Platform | -80°C | 16S rRNA gene sequencing (V3-V4) | NR |
| Zhang 2023-1^38^ | China | Stool | COVID(long COVID-19) | HC | 45/25 | 40.5/35.6 | 60/52 | Illumina Platform | -80°C | Metagenome sequenc_x005f ing | Yes |
| Covid-19 vs no Covid-19 control Respiratory tract | | | | |  |  |  |  |  |  |  |
| Maio 2020^46^ | Italy | Nasopharynx | COVID | No COVID | 18/22 | NR/NR | NR/NR | Illumina Platform |  | 16S rRNA gene sequencing （ V5–V6） | NR |
| Mostafa 2020^47^ | America | Nasopharyngeal Swab | COVID | No COVID | 40/10 | NR/NR | NR/NR | Nano Pore Gridion X5 | -80°C | Metagenomic sequencing | NR |
| Braun 2021^48^ | Israel | Nasopharyngeal | COVID | No COVID | 32/29 | 52/NR | 56/NR | Illumina Platform | NR | 16S rRNA gene sequencing (V4) | Yes |
| Engen 2021^49^ | America | Nasopharyngeal Swab | COVID | No COVID | 9/10 | 53.4/NR | 33.3/NR | Silva 138 | -80°C | 16S rRNA gene sequencing ( V4) | Yes |
| Gao 2021^50^ | China | Oropharyngeal | COVID | HC | 48/94 | 47/46 | 40/39.4 | Illumina Platform | -80°C | 16S rRNA gene sequencing ( V3–V4) | Yes |
| Gupta 2021-1^51^ | India | Nasopharyngeal Swabs | COVID | No COVID | 63/26 | 37.5/25.1 | 66.7/46.2 | Illumina Platform | NR | 16S rRNA gene sequencing (v4) | NR |
| Gupta 2021-2^52^ | India | Saliva | COVID | No COVID | 30/24 | 47/45 | 66.7/58.3 | Illumina Platform | -80°C | 16S rRNA gene sequencing ( V4) | Yes |
| Hernández‑Terán 2021^53^ | Mexico | Respiratory Samples | COVID(Mild) | HC | 18/7 | 37/35 | 51.4/28.6 | Illumina Platform | NR | 16S rRNA gene sequencing ( V3–V4) | NR |
|  |  |  | COVID(Severe) | HC | 27/7 | 47/35 | 56/28.6 |  |  |  |  |
|  |  |  | COVID(Fatal) | HC | 19/7 | 58/35 | 73.7/28.6 |  |  |  |  |
| Hoque 2021-1^54^ | Bangladesh | Nasopharyngeal Tract | COVID | HC | 8/7 | 39.6/48.1 | 75/71.4 | Illumina Platform | -80°C | Metagenome Sequencing | NR |
| Iebba 2021^55^ | Italy | Oral-Pharyngeal And Oral Swabs | COVID | HC | 26/15 | 67.3/NR | 80.8/NR | NR | NR | 16S rRNA gene sequencing ( V2–V3) | Yes |
| Ma 2021^56^ | China | Oropharynx Swab Specimens | COVID | HC | 31/28 | 50/37 | 54.8/42.9 | Mgiseq-2000 Platform | NR | Metagenome Sequencing | Yes |
| Nagy-Szakal 2021^57^ | America | Nasopharyngeal Swabs | COVID | No COVID | 26/22 | NR/NR | NR/NR | Illumina Platform | -70°C | Metagenomic sequencing analyses | NR |
| Nardelli 2021^58^ | Italy | Nasopharyngeal Swabs | COVID | No COVID | 18/12 | NR/NR | 66.7/33.3 | Illumina Platform | -80°C | 16S rRNA gene sequencing (V1-V2-V3) | NR |
| Ng 2021^59^ | America | Nasopharyngeal  Swab | COVID | No COVID | 137/108 | 49/44 | 48/26.5 | Illumina Platform | -80°C | Metatranscriptomic sequencing | NR |
| Rhoades 2021^60^ | America | Nasal | COVID | No COVID | 68/45 | 54.3/54.6 | 63.1/46.7 | Illumina Platform | NR | 16S rRNA gene sequencing (v5) | Yes |
| Ren 2021-1^10^ | China | Tongue Coating Samples | COVID | HC | 48/100 | 48.4/44.9 | 41.67/37 | Illumina Platform | -80°C | 16S rRNA gene sequencing (V3-V5) | NR |
| Ren 2021-2^61^ | China | Oropharyngeal Swab | COVID | HC | 192/95 | 58/47 | 59/40 | Illumina Platform | -80°C | Metatranscriptome  sequencing | Yes |
| Rosas-Salazar 2021^62^ | America | Upper Respiratory Tract | COVID | No COVID | 38/21 | 30.5/30 | 55.3/57.1 | Illumina Platform | NR | 16S rRNA gene sequencing (V4) | Yes |
| Rueca 2021 ^63^ | Italy | Nasal/Oropharyngeal Swabs | COVID(ICU) | HC | 10/10 | 57/53.5 | 60/30 | Ion S5 Sequencer | NR | 16S rRNA sequencing(V2–4–8, V3–6, V7–9) | Yes |
|  |  |  | COVID(Mild/ Moderate) | HC | 11/10 | 50/53.5 | 27.3/30 |  |  |  |  |
| Smith 2021^64^ | French | Nasopharynx Swabs | COVID(Moderate) | HC | 15/12 | NR/51 | NR/72 | Illumina Platform | NR | 16S rRNA sequencing, QPCR | Yes |
|  |  |  | COVID(Severe) | HC | 11/12 | NR/51 | NR/72 |  |  |  |  |
|  |  |  | COVID(critital) | HC | 23/12 | NR/51 | NR/72 |  |  |  |  |
| Soffritti 2021^65^ | Italy | Oral Rinse | COVID | No COVID | 39/36 | 71.1/66.5 | 51.3/61.1 | Ion Gene Studio S5 System | -80°C | Whole Genome Sequencing | Yes |
| Ventero1 2021^66^ | Spain | Nasopharyngeal Swab | COVID | No COVID | 56/18 | 64.2/48.1 | 58.9/50 | Illumina Platform | -80°C | 16S rRNA gene sequencing ( V3,V4) | Yes |
| Wu 2021^11^ | China | Throat Swab | COVID | HC | 52/44 | 48.5/41.5 | 57.7/70.5 | Illumina Platform | NR | 16S rRNA gene sequencing (V3-V5) | Yes |
| Xu 2021^12^ | China | Nasal Swabs | COVID | HC | 9/14 | 6.4/5.6 | 44.4/67.5 | Illumina Platform | NR | 16S rRNA gene sequencing ( V4) | Yes |
|  |  | Throat Swabs |  |  | 9/14 |  |  |  |  |  |  |
| Bai 2022^67^ | Sweden | Nasopharyngeal | COVID(Severe) | No COVID | 37/20 | 61/NR | 81.1/NR | Illumina Platform | NR | Metagenomic sequencing | Yes |
| Bradley 2022^68^ | America | Oropharyngeal Samples | COVID | No COVID | 74/41 | 68.1/65 | 48.7/71 | Illumina Platform | -20°C | Metagenome Sequencing |  |
| Callahan 2022^69^ | America | Saliva | COVID | No COVID | 16/90 | 47.2/52.3 | 43.8/40 | Illumina Platform | NR | 16S rRNA gene sequencing ( V1–V3) | Yes |
| Cui 2022^14^ | China | Tongue‑Coating | COVID | HC | 28/150 | 47.9/44.2 | 35.7/36.7 | Illumina Platform | -80°C | 16S rRNA gene sequencing ( V3-V5) | NR |
| Ferrari 2022^70^ | Italy | Nasopharyngeal Swabs | COVID | HC | 19/35 | NR/NR | NR/NR | Illumina Platform | -80°C | 16S rRNA gene sequencing (V3-V4) | Yes |
| Giugliano 2022^71^ | Italy | Nasopharynx Swabs | COVID | No COVID | 89/25 | 55/NR | 54/NR | Illumina Platform | NR | Next-Generation Sequencing | Yes |
| Gauthier 2022^72^ | China | Nasopharyngeal | COVID(Community-dwelling) | HC(Community-Dwelling) | 47/51 | 61.7/60.9 | 57.4/47.1 | Oxford Nanopore Technologies | NR | 16S rRNA gene sequencing | NR |
|  |  |  | COVID(Hospitalized ) | HC(Hospitalized ) | 48/48 | 64.1/61.9 | 68.8/68.8 |  |  |  |  |
| Hurst 2022^73^ | America | Nasopharyngeal | COVID | No COVID | 211/74 | NR/9.5 | 46.9/50 | Illumina Platform | -80°C | 16S rRNA gene sequencing (v4) | NR |
| Jitvaropas 2022^74^ | Thailand | Saliva | COVID(asymptomatic) | No COVID | 39/43 | 34.7/36.2 | 59/23 | Minion Mk1C | -80°C | 16S rRNA gene sequencing | NR |
| Kumar 2022^75^ | India | Nasopharyngeal Swabs | COVID(Deceased) | No COVID | 54/44 | 58/29 | 74.1/79.6 | Sub Systems Technology | NR | 16S rRNA gene sequencing ( V1–V3) | Yes |
| Paine 2022^76^ | India | Oropharyngeal Swabs | COVID | HC | 20/10 | 39.6/39.5 | 50/60 | Illumina Platform | isolated strictly within 4–6 h | 16S rRNA gene sequencing (V3-V4) | Yes |
| Prasad 2022^77^ | India | Nasopharyngeal | COVID(asymptomatic) | No COVID | 25/12 | 26/31 | 72/41.7 | Oxford Nanopore Long Read Sequencing Platform | -80°C | 16S rRNA gene sequencing ( V1–V9) | NR |
|  |  |  | COVID(Symptomatic) | No COVID | 21/12 | 32/31 | 90.5/41.7 |  |  |  |  |
|  |  |  | COVID | No COVID | 46/12 | NR/NR | 80.4/41.7 |  |  |  |  |
| Rattanaburi 2022^78^ | Thailand | Nasopharyngeal Swabs | COVID | HC | 24/24 | 36.1/34.5 | NR/NR | Illumina Platform | -80°C | 16S rRNA gene sequencing (V4) | NR |
| Rafiqul Islam 2022^22^ | Bangladesh | Saliva | COVID | HC | 22/15 | NR/NR | NR/NR | Illumina Platform | NR | 16S rRNA gene sequencing ( V3–V4) | Yes |
| Rocafort 2022^79^ | Spain | Nasopharyngeal | COVID | No COVID | 47/126 | 40/39.9 | 36.2/36.5 | Illumina Platform | NR | 16S rRNA gene sequencing ( V3–V4) | NR |
|  |  |  | COVID | No COVID | 45/425 | 4.7/4.4 | 51.1/52 |  |  |  |  |
| Shi 2022^80^ | China | Throat Swab | COVID(Mild) | HC | 10/10 | NR/NR | 50/NR | NR | -80°C | 16S rRNA gene sequencing | Yes |
| Shilts 2022^81^ | America | Upper Respiratory Tract | COVID(Mild) | No COVID | 27/20 | 49/31 | 56/60 | Illumina Platform | NR | 16S rRNA gene sequencing (V4) | Yes |
|  |  |  | COVID(Moderate) | No COVID | 28/20 | 33/31 | 46/60 |  |  |  |  |
|  |  |  | COVID(Severe) | No COVID | 15/20 | 58/31 | 33/60 |  |  |  |  |
| Tchoupou Saha 2022^82^ | French | Nasopharyngeal Pseudonymized | COVID | No COVID | 90/30 | 57.7/41.2 | 45.6/46.7 | Illumina Platform | NR | 16S rRNA gene sequencing ( V3–V4)， | NR |
| Zacharias 2022^83^ | Austria | Lung Tissue | COVID(Deceased) | Control(Deceased | 11/14 | 76.2/74.5 | 72.7/64.3 | Illumina Platform | -80°C | 16S rRNA gene sequencing (V4),Internal transcribed spacers sequence | Yes |
| Zhou 2022-2^43^ | China | Oropharyngeal samples | COVID | HC | 47/40 | 44.7/45.8 | 42.5/50 | Illumina Platform | NR | Metatranscriptomic Sequencing | NR |
| Al-Emran 2023^84^ | Bangladesh | Nasopharyngeal samples | COVID | No COVID | 4/4 | NR/NR | NR/NR | NR | NR | Metagenomic NGS sequencing | Yes |
| Alqedari 2023^85^ | America | Saliva samples | COVID(Mild or Moderate) | No COVID | 39/30 | NR/NR | 51/53 | Illumina Platform | -80°C | 16S rRNA gene sequencing (V4) | Yes |
|  |  |  | COVID(Severe) | No COVID | 11/30 | NR/NR | 73/53 |  |  |  |  |
| Leftwich 2023^34^ | America | Oral swabs | COVID | HC | 53/25 | NR/NR | 0/0 | Illumina Platform | NR | 16S rRNA gene sequencing (V3-V4) | NR |
| Hyblova 2023^86^ | Slovakia | Nasopharyngeal swab | COVID(Mild) | No COVID | 25/72 | 37/37 | 52/36.1 | Illumina Platform | -80°C | culture-independent RNA sequencing | Yes |
|  |  |  | COVID(Severe) | No COVID | 30/72 | 68/37 | 53.3/36.1 |  |  |  |  |
| Jiang 2023^87^ | China | Throat swab | COVID(Mild or Moderate) | HC | 49/37 | 74.5/57.7 | 61.2/43.2 | Illumina Platform | -80°C | 16S rRNA gene sequencing (V3-V4) | NR |
|  |  |  | COVID(Severe/Critical) | HC | 44/37 | 81.4/57.7 | 70.5/43.2 |  |  |  |  |
| Ling 2023^88^ | China | Nasopharyngeal swab and throat swab | COVID(Mild or Moderate) | HC | 106/15 | 49/42 | 56.6/66.7 | Illumina Platform | -80°C | 16S rRNA gene sequencing (V3-V4) | Yes |
|  |  |  | COVID(Severe/Critical) | HC | 51/15 | 66/42 | 31.4/66.7 |  |  |  |  |
| Lu 2023^89^ | China | Oropharyngeal microbiome | COVID | HC | 144/40 | NR/NR | 58.3/50 | Illumina Platform | -80°C | Metatranscriptomic analysis | Yes |
| Mahmud 2023^90^ | Bangladesh | Nasopharyngeal swab | COVID(Mild) | No COVID | 64/12 | 38.1/36.8 | 62.5/83.3 | Illumina Platform | -20°C | Shotgun metagenomic sequencing analyses | NR |
|  |  |  | COVID(Severe) | No COVID | 11/12 | 53.3/36.8 | 90.9/83.3 |  |  |  |  |
| Kim 2023^91^ | America | Saliva | COVID | No COVID | 60/18 | 54.5/44.9 | 66.7/55.6 | Illumina Platform | -80°C | 16S rRNA gene sequencing (V1-V2) | Yes |
|  |  | Nasopharyngeal Swab | COVID | No COVID | 54/12 | 64.3/57.6 | 55.6/33.3 |  |  |  |  |
| Wu 2023^92^ | China | Oropharyngeal swabs | COVID(Moderate) | HC | 30/24 | 58/57 | 50/58.3 | Illumina Platform | NR | 16S rRNA gene sequencing (V3-V4) | NR |
|  |  |  | COVID(Severe) | HC | 28/24 | 64.5/57 | 39.3/58.3 |  |  |  |  |
| Rosas-Salazar 2023^93^ | America | Upper respiratory tract samples | COVID | No COVID | 24/24 | 35.5/33 | 58.3/54.2 | Illumina Platform | NR | 16S rRNA gene sequencing (V4) | Yes |
| Yasir 2023^94^ | Korea | Nasopharyngeal Swab | COVID | No COVID | 85/22 | 44.2/38.4 | NR/NR | Illumina Platform | NR | 16S rRNA gene sequencing (V3-V4) | NR |
| Zhang 2023-1^38^ | China | Saliva  samples | COVID | HC | 45/25 | 40.5/35.6 | 60/52 | Illumina Platform | -80°C | Metagenome sequenc_x005f ing | Yes |
| Covid-19(recovered) vs no Covid-19 control Respiratory tract | | | | |  |  |  |  |  |  |  |
| Hoque 2021-1^54^ | Bangladesh | Nasopharyngeal Tract | COVID(Recovered) | HC | 7/7 | 35/48.1 | 57.1/71.4 | Illumina Platform | -80°C | Metagenome Sequencing | NR |
| Cui 2022^14^ | China | Tongue‑Coating | COVID(Recovered) | HC | 28/150 | 48.9/44.2 | 35.7/36.7 | Illumina Platform | -80°C | 16S rRNA gene sequencing ( V3-V5) | NR |
| Kumar 2022^75^ | India | Nasopharyngeal Swabs | COVID(Recovered) | No COVID | 43/44 | 35/29 | 74.4/79.6 | Sub Systems Technology | NR | 16S rRNA gene sequencing ( V1–V3) | Yes |
| Zhou 2022-2^43^ | China | Oropharyngeal samples | COVID(Recovered) | HC | 47/40 | 44.7/45.8 | 42.5/50 | Illumina Platform | NR | Metatranscriptomic Sequencing | NR |
| Wei 2023^95^ | China | Throat swab | COVID(Recovered) | HC | 23/29 | 36/37.6 | 73.9/51.7 | Illumina Platform | NR | 16S rRNA gene sequencing (V3-V4) | NR |
| Long Covid-19 vs no Covid-19 control Respiratory tract | | | | |  |  |  |  |  |  |  |
| Zhang 2023-1^38^ | China | Saliva  samples | COVID(long COVID-19,follow up) | HC | 45/25 | 40.5/35.6 | 60/52 | Illumina Platform | -80°C | Metagenome sequenc_x005f ing | Yes |
| Covid(severer) vs Covid-19(milder) Gut | | | | |  |  |  |  |  |  |  |
| Tang 2020^96^ | China | Stool | COVID(Severe) | COVID | 19/20 | 66/59 | 47.4/40 | NR | NR | QPCR | NR |
|  |  |  | COVID(Critical) | COVID | 18/20 | 68/59 | 66.7/40 | NR |  |  |  |
| Britton 2021^97^ | America | Stool | COVID(Moderate) | COVID(Mild) | 22/6 | NR/NR | NR/NR | Illumina Platform | -80°C | 16S rRNA gene sequencing (v4),Metagenome Sequencing | NR |
|  |  |  | COVID(Severe) | COVID(Mild) | 16/6 | NR/NR | NR/NR |  |  |  |  |
| Cao 2021^4^ | China | Stool | COVID(Severe) | COVID(Mild) | 3/3 | 66.7/34.3 | 66.7/66.7 | Illumina Platform | NR | Metagenomic sequencing analyses | Yes |
| Moreira-Rosário 2021^98^ | Portugal | Stool | COVID(Severe) | COVID(Mild) | 59/19 | 66/61 | 72.9/31.6 | Ion S5 Sequencer | -80°C | 16S rRNA gene sequencing(V3,V4) | Yes |
|  |  |  | COVID(Severe) | COVID(Moderate) | 59/37 | 66/71 | 72.9/64.9 |  |  |  |  |
| Reinold-2021^99^ | Germany | Rectal Swab | COVID(Severe/Critical) | COVID(No Severe) | 38/79 | 20/NR | 53/47 | NR | -80°C | 16S rRNA gene sequencing ( V3–V4) | Yes |
| Zhou 2021-2^100^ | China | Stool | COVID(Fever) | COVID(No Fever) | 127/60 | 37/48 | 36.2/31.7 | Mgi Seq 2000 | NR | Metagenome Sequencing | NR |
| Zuo 2021^101^ | China | Stool | COVID(High SARS-COV-2) | COVID(Low Sars-Cov-2) | 7/8 | NR/NR | NR/NR | Illumina Platform | NR | Shotgun metagenomic sequencing analyses | Yes |
| Albrich 2022^102^ | Switzerland | Stool | COVID(Severe) | COVID(Mild) | 89/42 | 65/58 | 80.9/38.1 | Illumina Platform | -80°C | 16S rRNA gene sequencing (V3,V4) | Yes |
|  |  |  | COVID(Deceased) | COVID(Mild) | 41/42 | 59.1/58 | 78/38.1 |  |  |  |  |
| Gan 2022^103^ | China | Stool | COVID(Severe) | COVID(Mild) | 4/19 | NR/NR | NR/NR | Mgiseq- 2000 Platform | NR | Metagenome Sequencing | Yes |
| Lai 2022^104^ | America | Stool | COVID(Severe) | COVID(Moderate) | 79/48 | 61/58.6 | 62/47.9 | Illumina Platform | -80°C | Metagenome Sequencing | Yes |
| Liu 2022-2^105^ | China | Stool | COVID(Severe) | COVID(No Severe) | 63/70 | 47.1/37.9 | 52.4/58.6 | Illumina Platform | -80°C | Metagenome Sequencing | yes |
| Maurer 2022^106^ | Germany | Stool | COVID(Aspergillus superinfection) | COVID(No Aspergillus Superinfection) | 16//26 | 76.5/61.5 | 69/58 | Illumina Platform | NR | 16S rRNA gene sequencing ( V3–V4) | NR |
| Mazzarelli 2022^107^ | Italy | Rectal Swab | COVID(Severe) | COVID(Mild) | 50/47 | 63/57.2 | 74/59.6 | Illumina Platform | -80°C | 16S rRNA gene sequencing(V3,V4) | NR |
| Shen 2022^108^ | China | Stool | COVID(ICU) | COVID(No Icu) | 20/46 | 70/51 | 80/56.5 | Illumina Platform | dry ice | Metagenome Sequencing | Yes |
| Sun 2022^26^ | China | Stool | COVID(Severe) | COVID(Mild) | 24/39 | 61/40 | 79.2/51.3 | Illumina Platform | -80°C | Metagenome Sequencing | Yes |
| Stutz 2022^109^ | America | Stool | COVID(Deceased) | COVID(Live) | 32/39 | 66.2/59 | 60/48.7 | Illumina Platform | -80°C | Metagenome Sequencing | Yes |
| Vestad 2022^110^ | Norway | Stool | COVID( respiratory dysfunction) | COVID(No Respiratory Dysfunction) | 25/58 | NR/NR | NR/NR | Illumina Platform | -80°C | 16S rRNA gene sequencing ( V3–V4) | NR |
| Yokoyama 2022^111^ | Japan | Stool | COVID(Critital) | COVID(No Critical) | 5/5 | 57.4/44.4 | 80/40 | Q2-Feature-Classifier | -80°C | 16S rRNA gene sequencing ( V3–V4) | NR |
| Guo 2023^112^ | China | Stool | COVID(Moderate) | COVID(Mild) | 196/88 | NR/NR | NR/NR | Illumina Platform | -80°C | Metagenomic sequencing | NR |
|  |  |  | COVID(Severe/Critical) | COVID(Mild) | 12/88 | NR/NR | NR/NR |  |  |  |  |
| Nagata 2023^36^ | Japan | Stool | COVID(Severe) | COVID(Mild) | 31/43 | NR/NR | NR/NR | Illumina Platform | -80°C | Metagenome Sequencing | Yes |
| Talukdar 2023^113^ | India | Stool | COVID(Severe) | COVID(Mild) | 45/7 | 60/45 | 71.1/85.7 | Illumina Platform | -20°C | 16S rRNA gene sequencing (V3-V4) | Yes |
| Trøseid 2023^114^ | Norway | Stool | COVID(ICU) | COVID(Ward) | 28/95 | 62/57 | 68/59 | Illumina Platform | -80°C | 16S rRNA gene sequencing (V3-V4) | NR |
| Covid(severer) vs Covid-19(milder) Respiratory tract | | | | |  |  |  |  |  |  |  |
| Sulaiman1 2021^115^ | America | Nasal Swab | COVID(decreased) | COVID( Mechanical Ventilation ≤28 Days) | 34/52 | 64/59 | 73.5/76.9 | Illumina Platform | DNA/RNA Shield | Metagenome and Metatranscriptome | Yes |
|  |  |  | COVID(decreased) | COVID( Mechanical Ventilation >28 Days) | 34/64 | 64/NR | 73.5/82.1 |  |  |  |  |
| Rosas-Salazar 2021^62^ | America | Upper Respiratory Tract | COVID(high SARS-CoV-2 vi V ral load) | COVID(Low Sars-Cov-2 Vir V Al Load) | NR/NR | NR/NR | NR/NR | Illumina Platform | NR | 16S rRNA gene sequencing (V4) | Yes |
| Chen 2022^116^ | China | Respiratory Tract | COVID(Severe) | COVID(Mild) | 28/41 | 71/57 | 57.1/34.1 | Illumina Platform | -80°C | 16S rRNA gene sequencing ( V3–V4) | NR |
| Devi 2022^117^ | India | Nasopharyngeal | COVID(Moderate) | COVID(Mild) | 36/24 | NR/NR | NR/NR | Illumina Platform | NR | Metatranscriptomic analysis | NR |
|  |  |  | COVID(Severe) | COVID(Mild) | 14/24 | NR/NR | NR/NR |  |  |  |  |
|  |  |  | COVID(Deceased) | COVID(Mild) | 12/24 | NR/NR | NR/NR |  |  |  |  |
| Gan 2022^103^ | China | Nasopharyngeal | COVID(Severe) | COVID(Mild) | 5/24 | NR/NR | NR/NR | Mgiseq- 2000 Platform | NR | Metagenome Sequencing | Yes |
|  |  | Oropharyngeal | COVID(Severe) | COVID(Mild) | 14/58 | NR/NR | NR/NR |  |  |  |  |
| Pozzi 2022^118^ | Italy | Saliva | COVID( hospitalized) | COVID(Non Hospitalized) | 25/23 | 68.2/41.4 | 76/34.8 | Illumina Platform | NR | 16S rRNA gene sequencing (V3-V4) | Yes |
| Qin 2022^119^ | China | Upper Respiratory Tracts | COVID(Severe) | COVID(Mild) | 51/20 | NR/NR | NR/NR | Illumina Platform | -80°C | 16S rRNA gene sequencing (V3-V4) | NR |
|  |  | Lower Respiratory Tracts | COVID(Severe) | COVID(Mild) | 51/20 | NR/NR | NR/NR |  |  |  |  |
| Shen 2022^108^ | China | Saliva | COVID(ICU) | COVID(No Icu) | 20/46 | 70/51 | 80/56.5 | Illumina Platform | dry ice | Metagenome Sequencing | Yes |
| Ventero1 2022^120^ | Spain | Nasopharyngeal | COVID(Deceased) | COVID(Live) | 31/146 | NR/NR | NR/NR | Illumina Platform | -80°C | 16S rRNA gene sequencing ( V3,V4) | NR |
| Alqedari 2023^85^ | America | Saliva samples | COVID(Severe) | COVID(No Severe) | 11/39 | NR/NR | 73/51 | Illumina Platform | -80°C | 16S rRNA gene sequencing (V4) | Yes |
| Larios Serrato 2023^121^ | America | Saliva samples | COVID(Severe) | COVID(Mild) | 57/103 | 50.8/41 | 66/43.5 | Illumina Platform | -70°C | 16S rRNA gene sequencing (V1-V3) | Yes |
|  |  |  | COVID(Deceased) | COVID(Mild) | 18/103 | 74.4/41 | 76.7/43.5 |  |  |  |  |
| Covid-19 vs no Covid-19 disease Gut | | | | |  |  |  |  |  |  |  |
| Gu 2020^1^ | China | Stool | COVID | H1N1 | 30/24 | 55/48.5 | 43.3/37.5 | Illumina Platform | -80°C | 16S rRNA gene sequencing ( V3-V4) | Yes |
| Mazzarelli 2021^122^ | Italy | Rectal Swab | COVID(ICU) | Non-COVID-19 Pneumonia | 6/8 | 70/69 | 50/62 | Ion 530 Chip | -80°C | 16S rRNA gene sequencing (V2, V4, V8 and V3-6, 7–9) | Yes |
|  |  |  | COVID(wards) | Non-COVID-19 Pneumonia | 9/8 | 67/69 | 55/62 |  |  |  |  |
| Newsome 2021^123^ | America | Stool | COVID | No COVID Patients | 50/34 | 62.3/55 | 56/41 | Illumina Platform | -80°C | 16S rRNA gene sequencing (V1-V3) | Yes |
| Reinold-2021^99^ | Germany | Rectal Swab | COVID | No COVID Patients | 117/95 | 56/50 | 49/52 | NR | -80°C | 16S rRNA gene sequencing ( V3–V4) | Yes |
| Cuenca 2022^15^ | Spain | Stool、Rectal Swab | COVID(ICU) | No COVID(Icu) | 46/32 | 61/58 | 70/32.5 | Illumina Platform | -80°C | 16S rRNA gene sequencing ( V4) | Yes |
| Romani 2022^23^ | Italy | Stool | COVID | No COVID Patients | 68/16 | 6.5/4.4 | 56/53 | Illumina Platform | -80°C | 16S rRNA gene sequencing (V3-V4) | Yes |
| Chen 2023^124^ | China | Stool | COVID | No COVID Patients | 27/22 | 54.9/45.4 | 40.7/50 | Ion Torrent  Proton Sequencer | NR | Metagenomic sequencing | Yes |
| Covid-19 vs no Covid-19 disease Respiratory tract | | | | |  |  |  |  |  |  |  |
| Gaibani 2021-2^125^ | Italy | Bronchoalveolar Lavage | COVID | Non-COVID-19 Pneumonia | 24/24 | 68/64 | 71/61 | Illumina Platform | NR | 16S rRNA gene sequencing (V3-V4) | Yes |
| Hoque 2021-2^126^ | Bangladesh | Nasopharyngeal | COVID | Copd, Upper Respiratory Tract Infection | 11/10 | NR/NR | NR/NR | Illumina Platform | -20°C | Metagenomic sequencing analyses | NR |
| Liu 2021^127^ | China | Nasopharyngeal Swabs | COVID | No COVID Patients | 9/6 | 38.9/45.3 | 44.1/76.7 | Illumina Platform | -80°C | Metagenomic sequencing | NR |
| Ma 2021^56^ | China | Oropharynx Swab Specimens | COVID | Influenza B | 31/29 | 50/59 | 54.8/69 | Mgiseq-2000 Platform | NR | Metagenome Sequencing | Yes |
| Merenstein 2021^128^ | America | Oropharyngeal | COVID | No COVID Patients | 83/13 | 64/60 | 53/69 | Illumina Platform | NR | 16S rRNA gene sequencing (V1,V2) | Yes |
|  |  | Nasopharyngeal |  |  |  |  |  |  |  |  |  |
| Miller 2021^129^ | America | Saliva | COVID | No COVID Patients | 46/54 | 56.5/56.2 | 45.3/61 | Illumina Platform | -80°C | 16S rRNA gene sequencing ( V1–V2) | Yes |
| Rhoades 2021^60^ | America | Nasal | COVID | No COVID Patients | 68/21 | 54.3/58.2 | 63.1/47.6 | Illumina Platform | NR | 16S rRNA gene sequencing (v4) | Yes |
| Xiong 2021^130^ | China | Pharyngeal Swabs | COVID | No COVID Patients | 11/11 | 45.6/52 | 36.4/45.5 | MGI-seq2000 platform | NR | Metagenome sequencing | NR |
| Zhang 2021^131^ | China | Nasopharyngeal Swabs | COVID | Non-COVID-19 Pneumonia | 24/36 | 47.3/47.2 | 54.2/47.2 | Illumina Platform | NR | RT-PCR，Metatranscriptomic sequencing | Yes |
|  |  | Sputum |  |  | 14/39 | 57.8/49.4 | 78.6/69.8 |  |  |  |  |
| Meng 2022^132^ | China | Nasopharyngeal Swabs | COVID | Non-COVID-19 pneumonia | 72/65 | NR/NR | NR/NR | Illumina Platform | -70°C | Metatranscriptomic sequencing | NR |
| Rattanaburi 2022^78^ | Thailand | Nasopharyngeal Swabs | COVID | Influenza A | 24/24 | 36.1/26 | NR/NR | Illumina Platform | -80°C | 16S rRNA gene sequencing (V4) | NR |
|  |  |  |  | Influenza B | 24/24 | 36.1/26.5 | NR/NR |  |  |  | NR |
| Javan 2023^133^ | America | Lung Tissue | COVID(Deceased) | No COVID Patients(Deceased) | 20/20 | 62.3/77.9 | 60/30 | Illumina Platform | -80°C | 16S rRNA gene sequencing (V4) | Yes |
| Xie 2023^134^ | China | Sputum and bronchoalveolar lavage fluid | COVID | Non COVID Pneumonia | 38/26 | 61.2/57 | 76.3/73.1 | Oxford Nanopore MinION | -80°C | Nanopore-targeted sequencing | Yes |

Supplementary table 2 Characteristic of included articles involving influenza

| First author & publication year | Country | Specimen | Disease | Control | Numbel (D/C) | Age (D/C) | Male % ( D/C) | Sequencing platform | Preservation condition | Microbiome assessment method | Multiple comparisons correction? |
| --- | --- | --- | --- | --- | --- | --- | --- | --- | --- | --- | --- |
|  |  |  |  |  |  |  |  |  |  |  |  |
| Influenza patients vs no Influenza patients Gut sample | | | | | |  |  |  |  |  |  |
| Qin 2015^135^ | China | Stool | H7N9(Antibiotic treatment) | HCs | 17/31 | 60.4/57.5 | 76.5/67.7 | Illumina platform | –80∘ C | Next generation sequencing | NR |
|  |  |  | H7N9(No Antibiotic treatment) | HCs | 9/31 | 51.6/57.5 | 66.7/67.7 |  |  |  |  |
| Gu 2020^1^ | China | Stool | HINI | HC | 24/30 | 48.5/53.5 | 37.5/43.3 | Illumina Platform | -80°C | 16S rRNA gene sequencing ( V3-V5) | Yes |
| Al Khatib 2021^136^ | Qatar | Stool | influenza | HCs | 38/11 | 31/34 | 100/100 | Miseq platform | –80∘ C | 16S rRNA gene sequence(V4) | NR |
| Fuentes 2021^137^ | Netherlands | Stool | Infuenza-like illness | no Infuenza-like illness control | 213/184 | 69.2/71.3 | 47/50 | Illumina platform | NR | 16S rRNA gene sequence(V4) | Yes |
| Influenza patients vs no Influenza patients Respiratory tract sample | | | | |  |  |  |  |  |  |  |
| Li 2017^138^ | China | Pharyngeal swab | HINI | HCs | 100/30 | 15.6/16.5 | 62/53.3 | Illumina platform | NR | 16S rRNA gene sequence(V3-V4) | NR |
|  |  |  | Non-H1N1 influenza | HCs | 72/30 | 13.4/16.5 | 58.3/53.3 |  |  |  |  |
| Lu 2017^139^ | China | oropharyngeal | H7N9(Secondary bacterial lung infection) | HCs | 21/30 | 60.5/50 | 42.9/36.7 | Illumina platform | NR | 16S rRNA gene sequence(V3-V4) | NR |
| Ramos-Sevillano 2018^140^ | United Kingdom | Nasopharyngeal swabs | Infuenza-like illness,influenza | HCs | 33/24 | NR/NR | NR/NR | Agilent | NR | 16S rRNA gene sequence(V1-V3) | Yes |
| Wen 2018^141^ | China | Nasopharyngeal | Infuenza A virus patients | HCs | 121/59 | 2.9/2.8 | 61.2/44.1 | Illumina platform | –80∘ C | 16S rRNA gene sequence(V3-V4) | Yes |
|  |  | oropharyngeal | Infuenza A virus patients | HCs | 121/59 | 2.9/2.8 | 61.2/44.1 |  |  |  |  |
| Ding 2019^142^ | America | nasopharyngeal | influenza | No influenza controls | 214/40 | 51.2/34.7 | 45.3/37.5 | Illumina platform | –20∘ C | 16S rRNA gene sequence(V4) | NR |
| Qin 2020^143^ | China | nasopharyngeal samples | influenza(mild) | HCs | 21/24 | 56.4/59 | 52.4/54 | Illumina platform | −70 ∘ C | 16S rRNA gene sequence(V3-V4) | Yes |
|  |  |  | influenza(severe) | HCs | 31/24 | 61.7/59 | 56.7/54 |  |  |  |  |
| Zhou 2020^144^ | China | nasopharyngea | influenza | HCs | 66/59 | NR/NR | NR/NR | NR | NR | 16S rRNA gene sequence(V3-V4) | NR |
|  |  | oropharyngeal | influenza | HCs | 66/59 | NR/NR | NR/NR |  |  |  |  |
| Ma 2021^56^ | China | oropharynx swab specimens | Influenza B | HC | 29/28 | 59/37 | 69/42.9 | MGISEQ-2000 platform | NR | Metagenome Sequencing | Yes |
| Hu 2022^145^ | China | Oropharyngeal swab | Influenza A virus pneumonia | HCs | 49/42 | 39/41.1 | 67.3/59.5 | Illumina platform | –80∘ C | 16S rRNA gene sequence(V3-V4) | Yes |
| Rattanaburi 2022^78^ | Thailand | Nasopharyngeal Swabs | influenza  A | HC | 24/24 | 26/34.5 | NR/NR | Illumina Platform | -80°C | 16S rRNA gene sequencing (V4) | NR |
|  |  |  | influenza  B | HC | 24/24 | 26.5/34.5 |  |  |  |  |  |
| Shetty 2022^146^ | Netherlands | Nasopharyngeal (NP) swabs | Infuenza-like illness | no Infuenza-like illness control | 240/157 | 69.5/71.4 | 47.9/51 | Illumina platform | NR | 16S rRNA gene sequence(V4) | Yes |
|  |  |  | H7N9(No Secondary bacterial lung infection) | HCs | 51/30 | 53/50 | 56.7/36.7 |  |  |  |  |
| Li 2023^147^ | China |  | influenza A | HCs | 22/98 | 29/31.5 | 50/8.2 | Illumina Platform | -80°C | 16S rRNA gene sequence(V4) | Yes |
|  |  |  | influenza B | HCs | 138/98 | 29/31.5 | 47.8/8.2 |  |  |  |  |
| Zhang 2023^148^ | America | nasal and throat swabs | influenza | No influenza controls | 42/93 | 6/14 | 52/20 | Illumina Platform | -80°C | metagenome  sequencing | Yes |
| Zhou 2023^149^ | China | bronchoalveolar lavage fuid | influenza | No influenza controls | 42/42 | 64.2/59.1 | 78.6/78.6 | Illumina Platform | -80°C | 16S rRNA gene sequence(V3-V4) | NR |

Supplementary table 3 The results of meta regression of Shannon index in gut samples ( COVID-19 patients versus no COVID-19 controls)

|  | estimate | se | zval | pval | ci.lb | ci.ub |
| --- | --- | --- | --- | --- | --- | --- |
| intrcpt | -0.4751 | 0.9704 | -0.4896 | 0.6245 | -2.3771 | 1.4269 |
| Region-Asian | -1.0599 | 1.0757 | -0.9854 | 0.3244 | -3.1682 | 1.0484 |
| Region-Europe | -0.6893 | 1.2634 | -0.5456 | 0.5854 | -3.1655 | 1.787 |
| Age-child | 1.0715 | 1.1009 | 0.9733 | 0.3304 | -1.0862 | 3.2291 |
| Type-mild | 0.9223 | 1.4586 | 0.6323 | 0.5272 | -1.9365 | 3.7812 |
| Type-severe | 0.3601 | 1.4541 | 0.2476 | 0.8044 | -2.4899 | 3.21 |

Supplementary table 4 The results of meta regression of Shannon index in respiratory tract sample ( COVID-19 patients versus no COVID-19 controls)

|  | estimate | se | zval | pval | ci.lb | ci.ub |
| --- | --- | --- | --- | --- | --- | --- |
| intrcpt | -0.3572 | 0.3736 | -0.9561 | 0.339 | -1.0894 | 0.375 |
| Region-Asian | -0.4778 | 0.4354 | -1.0974 | 0.2724 | -1.3312 | 0.3755 |
| Region-Europe | 0.0622 | 0.5174 | 0.1202 | 0.9043 | -0.9518 | 1.0762 |
| Age-child | 0.1588 | 1.021 | 0.1556 | 0.8764 | -1.8422 | 2.1599 |
| Type-critital | -1.0124 | 1.5453 | -0.6552 | 0.5124 | -4.0411 | 2.0163 |
| Type-mild | 0.5886 | 0.5394 | 1.0912 | 0.2752 | -0.4686 | 1.6457 |
| Type-moderate | 1.2177 | 0.8753 | 1.3912 | 0.1642 | -0.4978 | 2.9333 |
| Type-severe | -0.2277 | 0.4952 | -0.4599 | 0.6456 | -1.1982 | 0.7428 |

**References**

1. Gu S, Chen Y, Wu Z, et al. Alterations of the Gut Microbiota in Patients With Coronavirus Disease 2019 or H1N1 Influenza. *Clinical infectious diseases : an official publication of the Infectious Diseases Society of America* 2020;71(10):2669-78. doi: 10.1093/cid/ciaa709 [published Online First: 2020/06/05]

2. Zuo T, Zhang F, Lui GCY, et al. Alterations in Gut Microbiota of Patients With COVID-19 During Time of Hospitalization. *Gastroenterology* 2020;159(3):944-55.e8. doi: 10.1053/j.gastro.2020.05.048 [published Online First: 2020/05/23]

3. Al Bataineh MT, Henschel A, Mousa M, et al. Gut Microbiota Interplay With COVID-19 Reveals Links to Host Lipid Metabolism Among Middle Eastern Populations. *Frontiers in microbiology* 2021;12:761067. doi: 10.3389/fmicb.2021.761067 [published Online First: 2021/11/23]

4. Cao J, Wang C, Zhang Y, et al. Integrated gut virome and bacteriome dynamics in COVID-19 patients. *Gut microbes* 2021;13(1):1-21. doi: 10.1080/19490976.2021.1887722 [published Online First: 2021/03/09]

5. Gaibani P, D'Amico F, Bartoletti M, et al. The Gut Microbiota of Critically Ill Patients With COVID-19. *Frontiers in cellular and infection microbiology* 2021;11:670424. doi: 10.3389/fcimb.2021.670424 [published Online First: 2021/07/17]

6. He F, Zhang T, Xue K, et al. Fecal multi-omics analysis reveals diverse molecular alterations of gut ecosystem in COVID-19 patients. *Analytica chimica acta* 2021;1180:338881. doi: 10.1016/j.aca.2021.338881 [published Online First: 2021/09/21]

7. Kim HN, Joo EJ, Lee CW, et al. Reversion of Gut Microbiota during the Recovery Phase in Patients with Asymptomatic or Mild COVID-19: Longitudinal Study. *Microorganisms* 2021;9(6) doi: 10.3390/microorganisms9061237 [published Online First: 2021/07/03]

8. Khan M, Mathew BJ, Gupta P, et al. Gut Dysbiosis and IL-21 Response in Patients with Severe COVID-19. *Microorganisms* 2021;9(6) doi: 10.3390/microorganisms9061292 [published Online First: 2021/07/03]

9. Li S, Yang S, Zhou Y, et al. Microbiome Profiling Using Shotgun Metagenomic Sequencing Identified Unique Microorganisms in COVID-19 Patients With Altered Gut Microbiota. *Frontiers in microbiology* 2021;12:712081. doi: 10.3389/fmicb.2021.712081 [published Online First: 2021/10/29]

10. Ren Z, Wang H, Cui G, et al. Alterations in the human oral and gut microbiomes and lipidomics in COVID-19. *Gut* 2021;70(7):1253-65. doi: 10.1136/gutjnl-2020-323826 [published Online First: 2021/04/02]

11. Wu Y, Cheng X, Jiang G, et al. Altered oral and gut microbiota and its association with SARS-CoV-2 viral load in COVID-19 patients during hospitalization. *NPJ biofilms and microbiomes* 2021;7(1):61. doi: 10.1038/s41522-021-00232-5 [published Online First: 2021/07/24]

12. Xu R, Liu P, Zhang T, et al. Progressive deterioration of the upper respiratory tract and the gut microbiomes in children during the early infection stages of COVID-19. *Journal of genetics and genomics = Yi chuan xue bao* 2021;48(9):803-14. doi: 10.1016/j.jgg.2021.05.004 [published Online First: 2021/07/10]

13. Yeoh YK, Zuo T, Lui GC, et al. Gut microbiota composition reflects disease severity and dysfunctional immune responses in patients with COVID-19. *Gut* 2021;70(4):698-706. doi: 10.1136/gutjnl-2020-323020 [published Online First: 2021/01/13]

14. Cui GY, Rao BC, Zeng ZH, et al. Characterization of oral and gut microbiome and plasma metabolomics in COVID-19 patients after 1-year follow-up. *Military Medical Research* 2022;9(1):32. doi: 10.1186/s40779-022-00387-y [published Online First: 2022/06/18]

15. Cuenca S, Soler Z, Serrano-Gómez G, et al. Dysbiosis: An Indicator of COVID-19 Severity in Critically Ill Patients. *International journal of molecular sciences* 2022;23(24) doi: 10.3390/ijms232415808 [published Online First: 2022/12/24]

16. Hazan S, Stollman N, Bozkurt HS, et al. Lost microbes of COVID-19: Bifidobacterium, Faecalibacterium depletion and decreased microbiome diversity associated with SARS-CoV-2 infection severity. *BMJ open gastroenterology* 2022;9(1) doi: 10.1136/bmjgast-2022-000871 [published Online First: 2022/04/29]

17. Juárez-Castelán CJ, Vélez-Ixta JM, Corona-Cervantes K, et al. The Entero-Mammary Pathway and Perinatal Transmission of Gut Microbiota and SARS-CoV-2. *International journal of molecular sciences* 2022;23(18) doi: 10.3390/ijms231810306 [published Online First: 2022/09/24]

18. Liu Q, Mak JWY, Su Q, et al. Gut microbiota dynamics in a prospective cohort of patients with post-acute COVID-19 syndrome. *Gut* 2022;71(3):544-52. doi: 10.1136/gutjnl-2021-325989 [published Online First: 2022/01/28]

19. Maeda Y, Motooka D, Kawasaki T, et al. Longitudinal alterations of the gut mycobiota and microbiota on COVID-19 severity. *BMC infectious diseases* 2022;22(1):572. doi: 10.1186/s12879-022-07358-7 [published Online First: 2022/06/25]

20. Mizutani T, Ishizaka A, Koga M, et al. Correlation Analysis between Gut Microbiota Alterations and the Cytokine Response in Patients with Coronavirus Disease during Hospitalization. *Microbiology spectrum* 2022;10(2):e0168921. doi: 10.1128/spectrum.01689-21 [published Online First: 2022/03/08]

21. Nobre JG, Delgadinho M, Silva C, et al. Gut microbiota profile of COVID-19 patients: Prognosis and risk stratification (MicroCOVID-19 study). *Frontiers in microbiology* 2022;13:1035422. doi: 10.3389/fmicb.2022.1035422 [published Online First: 2022/12/10]

22. Rafiqul Islam SM, Foysal MJ, Hoque MN, et al. Dysbiosis of Oral and Gut Microbiomes in SARS-CoV-2 Infected Patients in Bangladesh: Elucidating the Role of Opportunistic Gut Microbes. *Frontiers in medicine* 2022;9:821777. doi: 10.3389/fmed.2022.821777 [published Online First: 2022/03/04]

23. Romani L, Del Chierico F, Macari G, et al. The Relationship Between Pediatric Gut Microbiota and SARS-CoV-2 Infection. *Frontiers in cellular and infection microbiology* 2022;12:908492. doi: 10.3389/fcimb.2022.908492 [published Online First: 2022/07/26]

24. Schult D, Reitmeier S, Koyumdzhieva P, et al. Gut bacterial dysbiosis and instability is associated with the onset of complications and mortality in COVID-19. *Gut microbes* 2022;14(1):2031840. doi: 10.1080/19490976.2022.2031840 [published Online First: 2022/02/18]

25. Suskun C, Kilic O, Yilmaz Ciftdogan D, et al. Intestinal microbiota composition of children with infection with severe acute respiratory syndrome coronavirus 2 (SARS-CoV-2) and multisystem inflammatory syndrome (MIS-C). *European journal of pediatrics* 2022;181(8):3175-91. doi: 10.1007/s00431-022-04494-9 [published Online First: 2022/05/19]

26. Sun Z, Song ZG, Liu C, et al. Gut microbiome alterations and gut barrier dysfunction are associated with host immune homeostasis in COVID-19 patients. *BMC medicine* 2022;20(1):24. doi: 10.1186/s12916-021-02212-0 [published Online First: 2022/01/21]

27. Upadhyay V, Suryawanshi RK, Tasoff P, et al. Mild SARS-CoV-2 infection results in long-lasting microbiota instability. *mBio* 2023:e0088923. doi: 10.1128/mbio.00889-23 [published Online First: 2023/06/09]

28. Xu X, Zhang W, Guo M, et al. Integrated analysis of gut microbiome and host immune responses in COVID-19. *Frontiers of medicine* 2022;16(2):263-75. doi: 10.1007/s11684-022-0921-6 [published Online First: 2022/03/09]

29. Yin YS, Minacapelli CD, Parmar V, et al. Alterations of the fecal microbiota in relation to acute COVID-19 infection and recovery. *Molecular biomedicine* 2022;3(1):36. doi: 10.1186/s43556-022-00103-1 [published Online First: 2022/11/28]

30. Zhang F, Wan Y, Zuo T, et al. Prolonged Impairment of Short-Chain Fatty Acid and L-Isoleucine Biosynthesis in Gut Microbiome in Patients With COVID-19. *Gastroenterology* 2022;162(2):548-61.e4. doi: 10.1053/j.gastro.2021.10.013 [published Online First: 2021/10/24]

31. Zhou T, Wu J, Zeng Y, et al. SARS-CoV-2 triggered oxidative stress and abnormal energy metabolism in gut microbiota. *MedComm* 2022;3(1):e112. doi: 10.1002/mco2.112 [published Online First: 2022/03/15]

32. Mannan A, Hoque MN, Noyon SH, et al. SARS-CoV-2 infection alters the gut microbiome in diabetes patients: A cross-sectional study from Bangladesh. J Med Virol 2023;95(4):e28691. doi: 10.1002/jmv.28691 [published Online First: 2023/03/23]

33. Gutiérrez-Díaz I, Sanz-Martinez M, Castro AM, et al. Microbial and immune faecal determinants in infants hospitalized with COVID-19 reflect bifidobacterial dysbiosis and immature intestinal immunity. European journal of pediatrics 2023 doi: 10.1007/s00431-023-05140-8 [published Online First: 2023/08/09]

34. Leftwich HK, Vargas-Robles D, Rojas-Correa M, et al. The microbiota of pregnant women with SARS-CoV-2 and their infants. Microbiome 2023;11(1):141. doi: 10.1186/s40168-023-01577-z [published Online First: 2023/06/27]

35. Mannan A, Hoque MN, Noyon SH, et al. SARS-CoV-2 infection alters the gut microbiome in diabetes patients: A cross-sectional study from Bangladesh. J Med Virol 2023;95(4):e28691. doi: 10.1002/jmv.28691 [published Online First: 2023/03/23]

36. Nagata N, Takeuchi T, Masuoka H, et al. Human Gut Microbiota and Its Metabolites Impact Immune Responses in COVID-19 and Its Complications. *Gastroenterology* 2023;164(2):272-88. doi: 10.1053/j.gastro.2022.09.024 [published Online First: 2022/09/27]

37. Wang YZ, Zhou JG, Lu YM, et al. Altered gut microbiota composition in children and their caregivers infected with the SARS-CoV-2 Omicron variant. World journal of pediatrics : WJP 2023;19(5):478-88. doi: 10.1007/s12519-022-00659-6 [published Online First: 2023/01/11]

38. Zhang D, Weng S, Xia C, et al. Gastrointestinal symptoms of long COVID-19 related to the ectopic colonization of specific bacteria that move between the upper and lower alimentary tract and alterations in serum metabolites. BMC medicine 2023;21(1):264. doi: 10.1186/s12916-023-02972-x [published Online First: 2023/07/20]

39.Tian Y, Sun KY, Meng TQ, et al. Gut Microbiota May Not Be Fully Restored in Recovered COVID-19 Patients After 3-Month Recovery. Frontiers in nutrition 2021;8:638825. doi: 10.3389/fnut.2021.638825 [published Online First: 2021/06/01]

40. Zhou Y, Zhang J, Zhang D, et al. Linking the gut microbiota to persistent symptoms in survivors of COVID-19 after discharge. *Journal of microbiology (Seoul, Korea)* 2021;59(10):941-48. doi: 10.1007/s12275-021-1206-5 [published Online First: 2021/08/13]

41. Ferreira-Junior AS, Borgonovi TF, De Salis LVV, et al. Detection of Intestinal Dysbiosis in Post-COVID-19 Patients One to Eight Months after Acute Disease Resolution. International journal of environmental research and public health 2022;19(16) doi: 10.3390/ijerph191610189 [published Online First: 2022/08/27]

42.Polo PG, Çolak-Al B, Sentürk H, et al. Gut bacteria after recovery from COVID-19: a pilot study. European review for medical and pharmacological sciences 2022;26(22):8599-611. doi: 10.26355/eurrev_202211_30397 [published Online First: 2022/12/03]

43. Zhou Y, Lu S, Wei X, et al. Metatranscriptomic Analysis Reveals Disordered Alterations in Oropharyngeal Microbiome during the Infection and Clearance Processes of SARS-CoV-2: A Warning for Secondary Infections. Biomolecules 2023;13(1) doi: 10.3390/biom13010006

44.Mańkowska-Wierzbicka D, Zuraszek J, Wierzbicka A, et al. Alterations in Gut Microbiota Composition in Patients with COVID-19: A Pilot Study of Whole Hypervariable 16S rRNA Gene Sequencing. Biomedicines 2023;11(2) doi: 10.3390/biomedicines11020367 [published Online First: 2023/02/26]

45. Zhang D, Zhou Y, Ma Y, et al. Gut Microbiota Dysbiosis Correlates With Long COVID-19 at One-Year After Discharge. J Korean Med Sci 2023;38(15):e120. doi: 10.3346/jkms.2023.38.e120 [published Online First: 2023/04/19]

46. De Maio F, Posteraro B, Ponziani FR, et al. Nasopharyngeal Microbiota Profiling of SARS-CoV-2 Infected Patients. *Biological procedures online* 2020;22:18. doi: 10.1186/s12575-020-00131-7 [published Online First: 2020/07/31]

47. Mostafa HH, Fissel JA, Fanelli B, et al. Metagenomic Next-Generation Sequencing of Nasopharyngeal Specimens Collected from Confirmed and Suspect COVID-19 Patients. mBio 2020;11(6) doi: 10.1128/mBio.01969-20 [published Online First: 2020/11/22]

48. Braun T, Halevi S, Hadar R, et al. SARS-CoV-2 does not have a strong effect on the nasopharyngeal microbial composition. Scientific reports 2021;11(1):8922. doi: 10.1038/s41598-021-88536-6 [published Online First: 2021/04/28]

49. Engen PA, Naqib A, Jennings C, et al. Nasopharyngeal Microbiota in SARS-CoV-2 Positive and Negative Patients. Biological procedures online 2021;23(1):10. doi: 10.1186/s12575-021-00148-6 [published Online First: 2021/06/02]

50. Gao M, Wang H, Luo H, et al. Characterization of the Human Oropharyngeal Microbiomes in SARS-CoV-2 Infection and Recovery Patients. Advanced science (Weinheim, Baden-Wurttemberg, Germany) 2021;8(20):e2102785. doi: 10.1002/advs.202102785 [published Online First: 2021/08/24]

51. Gupta A, Karyakarte R, Joshi S, et al. Nasopharyngeal microbiome reveals the prevalence of opportunistic pathogens in SARS-CoV-2 infected individuals and their association with host types. Microbes and infection 2022;24(1):104880. doi: 10.1016/j.micinf.2021.104880 [published Online First: 2021/08/24]

52. Gupta A, Bhanushali S, Sanap A, et al. Oral dysbiosis and its linkage with SARS-CoV-2 infection. Microbiological research 2022;261:127055. doi: 10.1016/j.micres.2022.127055 [published Online First: 2022/05/22]

53. Hernández-Terán A, Mejía-Nepomuceno F, Herrera MT, et al. Dysbiosis and structural disruption of the respiratory microbiota in COVID-19 patients with severe and fatal outcomes. Scientific reports 2021;11(1):21297. doi: 10.1038/s41598-021-00851-0 [published Online First: 2021/10/31]

54. Hoque MN, Sarkar MMH, Rahman MS, et al. SARS-CoV-2 infection reduces human nasopharyngeal commensal microbiome with inclusion of pathobionts. Scientific reports 2021;11(1):24042. doi: 10.1038/s41598-021-03245-4 [published Online First: 2021/12/17]

55. Iebba V, Zanotta N, Campisciano G, et al. Profiling of Oral Microbiota and Cytokines in COVID-19 Patients. Frontiers in microbiology 2021;12:671813. doi: 10.3389/fmicb.2021.671813 [published Online First: 2021/08/17]

56. Ma S, Zhang F, Zhou F, et al. Metagenomic analysis reveals oropharyngeal microbiota alterations in patients with COVID-19. Signal transduction and targeted therapy 2021;6(1):191. doi: 10.1038/s41392-021-00614-3 [published Online First: 2021/05/15]

57. Nagy-Szakal D, Couto-Rodriguez M, Wells HL, et al. Targeted Hybridization Capture of SARS-CoV-2 and Metagenomics Enables Genetic Variant Discovery and Nasal Microbiome Insights. Microbiology spectrum 2021;9(2):e0019721. doi: 10.1128/Spectrum.00197-21 [published Online First: 2021/09/02]

58. Nardelli C, Gentile I, Setaro M, et al. Nasopharyngeal Microbiome Signature in COVID-19 Positive Patients: Can We Definitively Get a Role to Fusobacterium periodonticum? Frontiers in cellular and infection microbiology 2021;11:625581. doi: 10.3389/fcimb.2021.625581 [published Online First: 2021/03/05]

59. Ng DL, Granados AC, Santos YA, et al. A diagnostic host response biosignature for COVID-19 from RNA profiling of nasal swabs and blood. Science advances 2021;7(6) doi: 10.1126/sciadv.abe5984 [published Online First: 2021/02/05]

60. Rhoades NS, Pinski AN, Monsibais AN, et al. Acute SARS-CoV-2 infection is associated with an increased abundance of bacterial pathogens, including Pseudomonas aeruginosa in the nose. Cell reports 2021;36(9):109637. doi: 10.1016/j.celrep.2021.109637 [published Online First: 2021/08/26]

61. Ren L, Wang Y, Zhong J, et al. Dynamics of the Upper Respiratory Tract Microbiota and Its Association with Mortality in COVID-19. American journal of respiratory and critical care medicine 2021;204(12):1379-90. doi: 10.1164/rccm.202103-0814OC [published Online First: 2021/09/18]

62. Rosas-Salazar C, Kimura KS, Shilts MH, et al. SARS-CoV-2 infection and viral load are associated with the upper respiratory tract microbiome. The Journal of allergy and clinical immunology 2021;147(4):1226-33.e2. doi: 10.1016/j.jaci.2021.02.001 [published Online First: 2021/02/13]

63. Rueca M, Fontana A, Bartolini B, et al. Investigation of Nasal/Oropharyngeal Microbial Community of COVID-19 Patients by 16S rDNA Sequencing. International journal of environmental research and public health 2021;18(4) doi: 10.3390/ijerph18042174 [published Online First: 2021/03/07]

64. Smith N, Goncalves P, Charbit B, et al. Distinct systemic and mucosal immune responses during acute SARS-CoV-2 infection. Nature immunology 2021;22(11):1428-39. doi: 10.1038/s41590-021-01028-7 [published Online First: 2021/09/03]

65. Soffritti I, D'Accolti M, Fabbri C, et al. Oral Microbiome Dysbiosis Is Associated With Symptoms Severity and Local Immune/Inflammatory Response in COVID-19 Patients: A Cross-Sectional Study. Frontiers in microbiology 2021;12:687513. doi: 10.3389/fmicb.2021.687513 [published Online First: 2021/07/13]

66. Ventero MP, Cuadrat RRC, Vidal I, et al. Nasopharyngeal Microbial Communities of Patients Infected With SARS-CoV-2 That Developed COVID-19. Frontiers in microbiology 2021;12:637430. doi: 10.3389/fmicb.2021.637430 [published Online First: 2021/04/06]

67. Bai X, Narayanan A, Skagerberg M, et al. Characterization of the Upper Respiratory Bacterial Microbiome in Critically Ill COVID-19 Patients. Biomedicines 2022;10(5) doi: 10.3390/biomedicines10050982 [published Online First: 2022/05/29]

68. Bradley ES, Zeamer AL, Bucci V, et al. Oropharyngeal microbiome profiled at admission is predictive of the need for respiratory support among COVID-19 patients. Frontiers in microbiology 2022;13:1009440. doi: 10.3389/fmicb.2022.1009440 [published Online First: 2022/10/18]

69. Callahan N, Hattar M, Barbour T, et al. Oral microbial taxa associated with risk for SARS-CoV-2 infection. Frontiers in oral health 2022;3:886341. doi: 10.3389/froh.2022.886341 [published Online First: 2022/09/20]

70. Ferrari L, Favero C, Solazzo G, et al. Nasopharyngeal Bacterial Microbiota Composition and SARS-CoV-2 IgG Antibody Maintenance in Asymptomatic/Paucisymptomatic Subjects. Frontiers in cellular and infection microbiology 2022;12:882302. doi: 10.3389/fcimb.2022.882302 [published Online First: 2022/07/26]

71. Giugliano R, Sellitto A, Ferravante C, et al. NGS analysis of nasopharyngeal microbiota in SARS-CoV-2 positive patients during the first year of the pandemic in the Campania Region of Italy. Microbial pathogenesis 2022;165:105506. doi: 10.1016/j.micpath.2022.105506 [published Online First: 2022/04/01]

72. Gauthier NPG, Locher K, MacDonald C, et al. Alterations in the nasopharyngeal microbiome associated with SARS-CoV-2 infection status and disease severity. PloS one 2022;17(10):e0275815. doi: 10.1371/journal.pone.0275815 [published Online First: 2022/10/15]

73. Hurst JH, McCumber AW, Aquino JN, et al. Age-Related Changes in the Nasopharyngeal Microbiome Are Associated With Severe Acute Respiratory Syndrome Coronavirus 2 (SARS-CoV-2) Infection and Symptoms Among Children, Adolescents, and Young Adults. Clinical infectious diseases : an official publication of the Infectious Diseases Society of America 2022;75(1):e928-e37. doi: 10.1093/cid/ciac184 [published Online First: 2022/03/06]

74. Jitvaropas R, Mayuramart O, Sawaswong V, et al. Classification of salivary bacteriome in asymptomatic COVID-19 cases based on long-read nanopore sequencing. Experimental biology and medicine (Maywood, NJ) 2022;247(21):1937-46. doi: 10.1177/15353702221118091 [published Online First: 2022/09/10]

75. Kumar D, Pandit R, Sharma S, et al. Nasopharyngeal microbiome of COVID-19 patients revealed a distinct bacterial profile in deceased and recovered individuals. Microbial pathogenesis 2022;173(Pt A):105829. doi: 10.1016/j.micpath.2022.105829 [published Online First: 2022/10/18]

76. Paine SK, Rout UK, Bhattacharyya C, et al. Temporal dynamics of oropharyngeal microbiome among SARS-CoV-2 patients reveals continued dysbiosis even after Viral Clearance. NPJ biofilms and microbiomes 2022;8(1):67. doi: 10.1038/s41522-022-00330-y [published Online First: 2022/08/25]

77. Prasad P, Mahapatra S, Mishra R, et al. Long-read 16S-seq reveals nasopharynx microbial dysbiosis and enrichment of Mycobacterium and Mycoplasma in COVID-19 patients: a potential source of co-infection. Molecular omics 2022;18(6):490-505. doi: 10.1039/d2mo00044j [published Online First: 2022/05/05]

78. Rattanaburi S, Sawaswong V, Chitcharoen S, et al. Bacterial microbiota in upper respiratory tract of COVID-19 and influenza patients. Experimental biology and medicine (Maywood, NJ) 2022;247(5):409-15. doi: 10.1177/15353702211057473 [published Online First: 2021/11/16]

79. Rocafort M, Henares D, Brotons P, et al. Impact of COVID-19 Lockdown on the Nasopharyngeal Microbiota of Children and Adults Self-Confined at Home. Viruses 2022;14(7) doi: 10.3390/v14071521 [published Online First: 2022/07/28]

80. Shi YL, He MZ, Han MZ, et al. Characterization of Altered Oropharyngeal Microbiota in Hospitalized Patients With Mild SARS-CoV-2 Infection. Frontiers in cellular and infection microbiology 2022;12:824578. doi: 10.3389/fcimb.2022.824578 [published Online First: 2022/04/05]

81. Shilts MH, Rosas-Salazar C, Strickland BA, et al. Severe COVID-19 Is Associated With an Altered Upper Respiratory Tract Microbiome. Frontiers in cellular and infection microbiology 2021;11:781968. doi: 10.3389/fcimb.2021.781968 [published Online First: 2022/02/11]

82. Tchoupou Saha OF, Dubourg G, Yacouba A, et al. Profile of the Nasopharyngeal Microbiota Affecting the Clinical Course in COVID-19 Patients. *Frontiers in microbiology* 2022;13:871627. doi: 10.3389/fmicb.2022.871627 [published Online First: 2022/06/04]

83. Zacharias M, Kashofer K, Wurm P, et al. Host and microbiome features of secondary infections in lethal covid-19. iScience 2022;25(9):104926. doi: 10.1016/j.isci.2022.104926 [published Online First: 2022/08/23]

84. Al-Emran HM, Rahman S, Hasan MS, et al. Microbiome analysis revealing microbial interactions and secondary bacterial infections in COVID-19 patients comorbidly affected by Type 2 diabetes. J Med Virol 2023;95(1):e28234. doi: 10.1002/jmv.28234 [published Online First: 2022/10/19]

85. Alqedari H, Altabtbaei K, Espinoza JL, et al. Host-Microbiome Associations in Saliva Predict COVID-19 Severity. bioRxiv : the preprint server for biology 2023 doi: 10.1101/2023.05.02.539155 [published Online First: 2023/05/19]

86. Hyblova M, Hadzega D, Babisova K, et al. Metatranscriptome Analysis of Nasopharyngeal Swabs across the Varying Severity of COVID-19 Disease Demonstrated Unprecedented Species Diversity. Microorganisms 2023;11(7) doi: 10.3390/microorganisms11071804 [published Online First: 2023/07/29]

87. Jiang Z, Yang L, Qian X, et al. Tongue coating microbiome composition reflects disease severity in patients with COVID-19 in Nanjing, China. Journal of oral microbiology 2023;15(1) doi: 10.1080/20002297.2023.2236429

88. Ling L, Lai CKC, Lui G, et al. Characterization of upper airway microbiome across severity of COVID-19 during hospitalization and treatment. Frontiers in cellular and infection microbiology 2023;13:1205401. doi: 10.3389/fcimb.2023.1205401 [published Online First: 2023/07/20]

89. Lu S, Zhou Y, Hu Y, et al. Metatranscriptomic analysis revealed Prevotella as a potential biomarker of oropharyngeal microbiomes in SARS-CoV-2 infection. Frontiers in cellular and infection microbiology 2023;13 doi: 10.3389/fcimb.2023.1161763

90. Mahmud ASM, Seers CA, Shaikh AA, et al. A multicentre study reveals dysbiosis in the microbial co-infection and antimicrobial resistance gene profile in the nasopharynx of COVID-19 patients. Scientific reports 2023;13(1):4122. doi: 10.1038/s41598-023-30504-3 [published Online First: 2023/03/15]

91. Kim JG, Zhang A, Rauseo AM, et al. The salivary and nasopharyngeal microbiomes are associated with SARS-CoV-2 infection and disease severity. Journal of medical virology 2023;95(2):e28445. doi: 10.1002/jmv.28445 [published Online First: 2022/12/31]

92. Wu J, Liu W, Zhu L, et al. Dysbiosis of oropharyngeal microbiome and antibiotic resistance in hospitalized COVID-19 patients. J Med Virol 2023;95(4):e28727. doi: 10.1002/jmv.28727 [published Online First: 2023/05/15]

93. Rosas-Salazar C, Kimura KS, Shilts MH, et al. Upper respiratory tract microbiota dynamics following COVID-19 in adults. Microbial genomics 2023;9(2) doi: 10.1099/mgen.0.000957

94. Yasir M, Al-Sharif HA, Al-Subhi T, et al. Analysis of the nasopharyngeal microbiome and respiratory pathogens in COVID-19 patients from Saudi Arabia. Journal of infection and public health 2023;16(5):680-88. doi: 10.1016/j.jiph.2023.03.001 [published Online First: 2023/03/20]

95. Wei N, Zhu G, Zhao T, et al. Characterization of oral bacterial and fungal microbiome in recovered COVID-19 patients. BMC microbiology 2023;23(1):123. doi: 10.1186/s12866-023-02872-3 [published Online First: 2023/05/09]

96. Tang L, Gu S, Gong Y, et al. Clinical Significance of the Correlation between Changes in the Major Intestinal Bacteria Species and COVID-19 Severity. Engineering (Beijing, China) 2020;6(10):1178-84. doi: 10.1016/j.eng.2020.05.013 [published Online First: 2021/02/02]

97. Britton GJ, Chen-Liaw A, Cossarini F, et al. Limited intestinal inflammation despite diarrhea, fecal viral RNA and SARS-CoV-2-specific IgA in patients with acute COVID-19. Scientific reports 2021;11(1):13308. doi: 10.1038/s41598-021-92740-9 [published Online First: 2021/06/27]

98. Moreira-Rosário A, Marques C, Pinheiro H, et al. Gut Microbiota Diversity and C-Reactive Protein Are Predictors of Disease Severity in COVID-19 Patients. Frontiers in microbiology 2021;12:705020. doi: 10.3389/fmicb.2021.705020 [published Online First: 2021/08/06]

99. Reinold J, Farahpour F, Fehring C, et al. A Pro-Inflammatory Gut Microbiome Characterizes SARS-CoV-2 Infected Patients and a Reduction in the Connectivity of an Anti-Inflammatory Bacterial Network Associates With Severe COVID-19. Frontiers in cellular and infection microbiology 2021;11:747816. doi: 10.3389/fcimb.2021.747816 [published Online First: 2021/12/07]

100. Zhou Y, Shi X, Fu W, et al. Gut Microbiota Dysbiosis Correlates with Abnormal Immune Response in Moderate COVID-19 Patients with Fever. Journal of inflammation research 2021;14:2619-31. doi: 10.2147/jir.S311518 [published Online First: 2021/06/26]

101. Zuo T, Liu Q, Zhang F, et al. Depicting SARS-CoV-2 faecal viral activity in association with gut microbiota composition in patients with COVID-19. *Gut* 2021;70(2):276-84. doi: 10.1136/gutjnl-2020-322294 [published Online First: 2020/07/22]

102. Albrich WC, Ghosh TS, Ahearn-Ford S, et al. A high-risk gut microbiota configuration associates with fatal hyperinflammatory immune and metabolic responses to SARS-CoV-2. Gut microbes 2022;14(1):2073131. doi: 10.1080/19490976.2022.2073131 [published Online First: 2022/05/17]

103. Gan H, Min J, Long H, et al. Microbial and human transcriptional profiling of coronavirus disease 2019 patients: Potential predictors of disease severity. Frontiers in microbiology 2022;13:959433. doi: 10.3389/fmicb.2022.959433 [published Online First: 2022/09/20]

104. Lai P, Nguyen L, Okin D, et al. Metagenomic assessment of gut microbial communities and risk of severe COVID-19. Research square 2022 doi: 10.21203/rs.3.rs-1717624/v1 [published Online First: 2022/06/10]

105. Liu Q, Su Q, Zhang F, et al. Multi-kingdom gut microbiota analyses define COVID-19 severity and post-acute COVID-19 syndrome. Nature communications 2022;13(1):6806. doi: 10.1038/s41467-022-34535-8 [published Online First: 2022/11/11]

106. Maurer HC, Schult D, Koyumdzhieva P, et al. Gut Microbial Disruption in Critically Ill Patients with COVID-19-Associated Pulmonary Aspergillosis. Journal of fungi (Basel, Switzerland) 2022;8(12) doi: 10.3390/jof8121265 [published Online First: 2022/12/23]

107. Mazzarelli A, Giancola ML, Fontana A, et al. Gut microbiota composition in COVID-19 hospitalized patients with mild or severe symptoms. Frontiers in microbiology 2022;13:1049215. doi: 10.3389/fmicb.2022.1049215 [published Online First: 2022/12/24]

108. Shen Y, Yu F, Zhang D, et al. Dynamic Alterations in the Respiratory Tract Microbiota of Patients with COVID-19 and its Association with Microbiota in the Gut. Advanced science (Weinheim, Baden-Wurttemberg, Germany) 2022;9(27):e2200956. doi: 10.1002/advs.202200956 [published Online First: 2022/07/04]

109. Stutz MR, Dylla NP, Pearson SD, et al. Immunomodulatory fecal metabolites are associated with mortality in COVID-19 patients with respiratory failure. Nature communications 2022;13(1):6615. doi: 10.1038/s41467-022-34260-2 [published Online First: 2022/11/05]

110. Vestad B, Ueland T, Lerum TV, et al. Respiratory dysfunction three months after severe COVID-19 is associated with gut microbiota alterations. Journal of internal medicine 2022;291(6):801-12. doi: 10.1111/joim.13458 [published Online First: 2022/02/26]

111. Yokoyama Y, Ichiki T, Yamakawa T, et al. Impaired tryptophan metabolism in the gastrointestinal tract of patients with critical coronavirus disease 2019. Frontiers in medicine 2022;9:941422. doi: 10.3389/fmed.2022.941422 [published Online First: 2022/08/30]

112. Guo M, Wu G, Tan Y, et al. Guild-Level Microbiome Signature Associated with COVID-19 Severity and Prognosis. mBio 2023;14(1) doi: 10.1128/mbio.03519-22

113. Talukdar D, Bandopadhyay P, Ray Y, et al. Association of gut microbial dysbiosis with disease severity, response to therapy and disease outcomes in Indian patients with COVID-19. Gut pathogens 2023;15(1):22. doi: 10.1186/s13099-023-00546-z [published Online First: 2023/05/10]

114. Trøseid M, Holter JC, Holm K, et al. Gut microbiota composition during hospitalization is associated with 60-day mortality after severe COVID-19. Critical care (London, England) 2023;27(1):69. doi: 10.1186/s13054-023-04356-2 [published Online First: 2023/02/24]

115. Sulaiman I, Chung M, Angel L, et al. Microbial signatures in the lower airways of mechanically ventilated COVID-19 patients associated with poor clinical outcome. Nature microbiology 2021;6(10):1245-58. doi: 10.1038/s41564-021-00961-5 [published Online First: 2021/09/02]

116. Chen J, Liu X, Liu W, et al. Comparison of the respiratory tract microbiome in hospitalized COVID-19 patients with different disease severity. Journal of medical virology 2022;94(11):5284-93. doi: 10.1002/jmv.28002 [published Online First: 2022/07/16]

117. Devi P, Maurya R, Mehta P, et al. Increased Abundance of Achromobacter xylosoxidans and Bacillus cereus in Upper Airway Transcriptionally Active Microbiome of COVID-19 Mortality Patients Indicates Role of Co-Infections in Disease Severity and Outcome. Microbiology spectrum 2022;10(3):e0231121. doi: 10.1128/spectrum.02311-21 [published Online First: 2022/05/18]

118. Pozzi C, Levi R, Braga D, et al. A 'Multiomic' Approach of Saliva Metabolomics, Microbiota, and Serum Biomarkers to Assess the Need of Hospitalization in Coronavirus Disease 2019. Gastro hep advances 2022;1(2):194-209. doi: 10.1016/j.gastha.2021.12.006 [published Online First: 2022/02/18]

119. Qin T, Wang Y, Deng J, et al. Super Dominant Pathobiontic Bacteria in the Nasopharyngeal Microbiota Cause Secondary Bacterial Infection in COVID-19 Patients. Microbiology spectrum 2022;10(3):e0195621. doi: 10.1128/spectrum.01956-21 [published Online First: 2022/05/18]

120. Ventero MP, Moreno-Perez O, Molina-Pardines C, et al. Nasopharyngeal Microbiota as an early severity biomarker in COVID-19 hospitalised patients. The Journal of infection 2022;84(3):329-36. doi: 10.1016/j.jinf.2021.12.030 [published Online First: 2021/12/30]

121. Larios Serrato V, Meza B, Gonzalez-Torres C, et al. Diversity, composition, and networking of saliva microbiota distinguish the severity of COVID-19 episodes as revealed by an analysis of 16S rRNA variable V1-V3 region sequences. mSystems 2023:e0106222. doi: 10.1128/msystems.01062-22 [published Online First: 2023/06/13]

122. Mazzarelli A, Giancola ML, Farina A, et al. 16S rRNA gene sequencing of rectal swab in patients affected by COVID-19. PloS one 2021;16(2):e0247041. doi: 10.1371/journal.pone.0247041 [published Online First: 2021/02/18]

123. Newsome RC, Gauthier J, Hernandez MC, et al. The gut microbiome of COVID-19 recovered patients returns to uninfected status in a minority-dominated United States cohort. Gut microbes 2021;13(1):1-15. doi: 10.1080/19490976.2021.1926840 [published Online First: 2021/06/09]

124. Chen Y, Li X, Yu C, et al. Gut microbiome alterations in patients with COVID-19-related coagulopathy. Annals of hematology 2023;102(6):1589-98. doi: 10.1007/s00277-023-05186-6 [published Online First: 2023/04/12]

125. Gaibani P, Viciani E, Bartoletti M, et al. The lower respiratory tract microbiome of critically ill patients with COVID-19. Scientific reports 2021;11(1):10103. doi: 10.1038/s41598-021-89516-6 [published Online First: 2021/05/14]

126. Hoque MN, Rahman MS, Ahmed R, et al. Diversity and genomic determinants of the microbiomes associated with COVID-19 and non-COVID respiratory diseases. Gene reports 2021;23:101200. doi: 10.1016/j.genrep.2021.101200 [published Online First: 2021/05/13]

127. Liu J, Liu S, Zhang Z, et al. Association between the nasopharyngeal microbiome and metabolome in patients with COVID-19. Synthetic and systems biotechnology 2021;6(3):135-43. doi: 10.1016/j.synbio.2021.06.002 [published Online First: 2021/06/22]

128. Merenstein C, Liang G, Whiteside SA, et al. Signatures of COVID-19 Severity and Immune Response in the Respiratory Tract Microbiome. mBio 2021;12(4):e0177721. doi: 10.1128/mBio.01777-21 [published Online First: 2021/08/18]

129. Miller EH, Annavajhala MK, Chong AM, et al. Oral Microbiome Alterations and SARS-CoV-2 Saliva Viral Load in Patients with COVID-19. Microbiology spectrum 2021;9(2):e0005521. doi: 10.1128/Spectrum.00055-21 [published Online First: 2021/10/14]

130. Xiong D, Muema C, Zhang X, et al. Enriched Opportunistic Pathogens Revealed by Metagenomic Sequencing Hint Potential Linkages between Pharyngeal Microbiota and COVID-19. Virologica Sinica 2021;36(5):924-33. doi: 10.1007/s12250-021-00391-x [published Online First: 2021/05/13]

131. Zhang H, Ai JW, Yang W, et al. Metatranscriptomic Characterization of Coronavirus Disease 2019 Identified a Host Transcriptional Classifier Associated With Immune Signaling. Clinical infectious diseases : an official publication of the Infectious Diseases Society of America 2021;73(3):376-85. doi: 10.1093/cid/ciaa663 [published Online First: 2020/05/29]

132. Meng H, Wang S, Tang X, et al. Respiratory immune status and microbiome in recovered COVID-19 patients revealed by metatranscriptomic analyses. Frontiers in cellular and infection microbiology 2022;12:1011672. doi: 10.3389/fcimb.2022.1011672 [published Online First: 2022/12/10]

133. Javan GT, Finley SJ, Moretti M, et al. COVID-19 and brain-heart-lung microbial fingerprints in Italian cadavers. Frontiers in molecular biosciences 2023;10:1196328. doi: 10.3389/fmolb.2023.1196328 [published Online First: 2023/06/30]

134. Xie L, Chen L, Li X, et al. Analysis of Lung Microbiome in COVID-19 Patients during Time of Hospitalization. Pathogens (Basel, Switzerland) 2023;12(7) doi: 10.3390/pathogens12070944 [published Online First: 2023/07/29]

135. Qin N, Zheng B, Yao J, et al. Influence of H7N9 virus infection and associated treatment on human gut microbiota. Scientific reports 2015;5:14771. doi: 10.1038/srep14771 [published Online First: 2015/10/23]

136. Al Khatib HA, Mathew S, Smatti MK, et al. Profiling of Intestinal Microbiota in Patients Infected with Respiratory Influenza A and B Viruses. Pathogens (Basel, Switzerland) 2021;10(6) doi: 10.3390/pathogens10060761 [published Online First: 2021/07/03]

137. Fuentes S, den Hartog G, Nanlohy NM, et al. Associations of faecal microbiota with influenza-like illness in participants aged 60 years or older: an observational study. The lancet Healthy longevity 2021;2(1):e13-e23. doi: 10.1016/s2666-7568(20)30034-9 [published Online First: 2021/01/01]

138. Li Y, Ding J, Xiao Y, et al. 16S rDNA sequencing analysis of upper respiratory tract flora in patients with influenza H1N1 virus infection. Frontiers in Laboratory Medicine 2017;1(1):16-26. doi: 10.1016/j.flm.2017.02.005

139. Lu HF, Li A, Zhang T, et al. Disordered oropharyngeal microbial communities in H7N9 patients with or without secondary bacterial lung infection. Emerging microbes & infections 2017;6(12):e112. doi: 10.1038/emi.2017.101 [published Online First: 2017/12/21]

140. Ramos-Sevillano E, Wade WG, Mann A, et al. The Effect of Influenza Virus on the Human Oropharyngeal Microbiome. Clinical infectious diseases : an official publication of the Infectious Diseases Society of America 2019;68(12):1993-2002. doi: 10.1093/cid/ciy821 [published Online First: 2018/11/18]

141. Wen Z, Xie G, Zhou Q, et al. Distinct Nasopharyngeal and Oropharyngeal Microbiota of Children with Influenza A Virus Compared with Healthy Children. BioMed research international 2018;2018:6362716. doi: 10.1155/2018/6362716 [published Online First: 2018/12/26]

142. Ding T, Song T, Zhou B, et al. Microbial Composition of the Human Nasopharynx Varies According to Influenza Virus Type and Vaccination Status. mBio 2019;10(4) doi: 10.1128/mBio.01296-19 [published Online First: 2019/07/04]

143. Qin T, Geng T, Zhou H, et al. Super-dominant pathobiontic bacteria in the nasopharyngeal microbiota as causative agents of secondary bacterial infection in influenza patients. Emerging microbes & infections 2020;9(1):605-15. doi: 10.1080/22221751.2020.1737578 [published Online First: 2020/03/18]

144. Zhou Q, Xie G, Liu Y, et al. Different nasopharynx and oropharynx microbiota imbalance in children with Mycoplasma pneumoniae or influenza virus infection. Microbial pathogenesis 2020;144:104189. doi: 10.1016/j.micpath.2020.104189 [published Online First: 2020/04/13]

145. Hu Q, Liu B, Fan Y, et al. Multi-omics association analysis reveals interactions between the oropharyngeal microbiome and the metabolome in pediatric patients with influenza A virus pneumonia. Frontiers in cellular and infection microbiology 2022;12:1011254. doi: 10.3389/fcimb.2022.1011254 [published Online First: 2022/11/18]

146. Shetty SA, van Beek J, Bijvank E, et al. Associations and recovery dynamics of the nasopharyngeal microbiota during influenza-like illness in the aging population. Scientific reports 2022;12(1):1915. doi: 10.1038/s41598-022-05618-9 [published Online First: 2022/02/05]

147. Li H, Wu X, Zeng H, et al. Unique microbial landscape in the human oropharynx during different types of acute respiratory tract infections. Microbiome 2023;11(1) doi: 10.1186/s40168-023-01597-9

148. Zhang L, Rahman J, Chung M, et al. CRISPR arrays as high-resolution markers to track microbial transmission during influenza infection. Microbiome 2023;11(1) doi: 10.1186/s40168-023-01568-0

149. Zhou Y, Du J, Wu JQ, et al. Impact of influenza virus infection on lung microbiome in adults with severe pneumonia. Annals of clinical microbiology and antimicrobials 2023;22(1) doi: 10.1186/s12941-023-00590-2


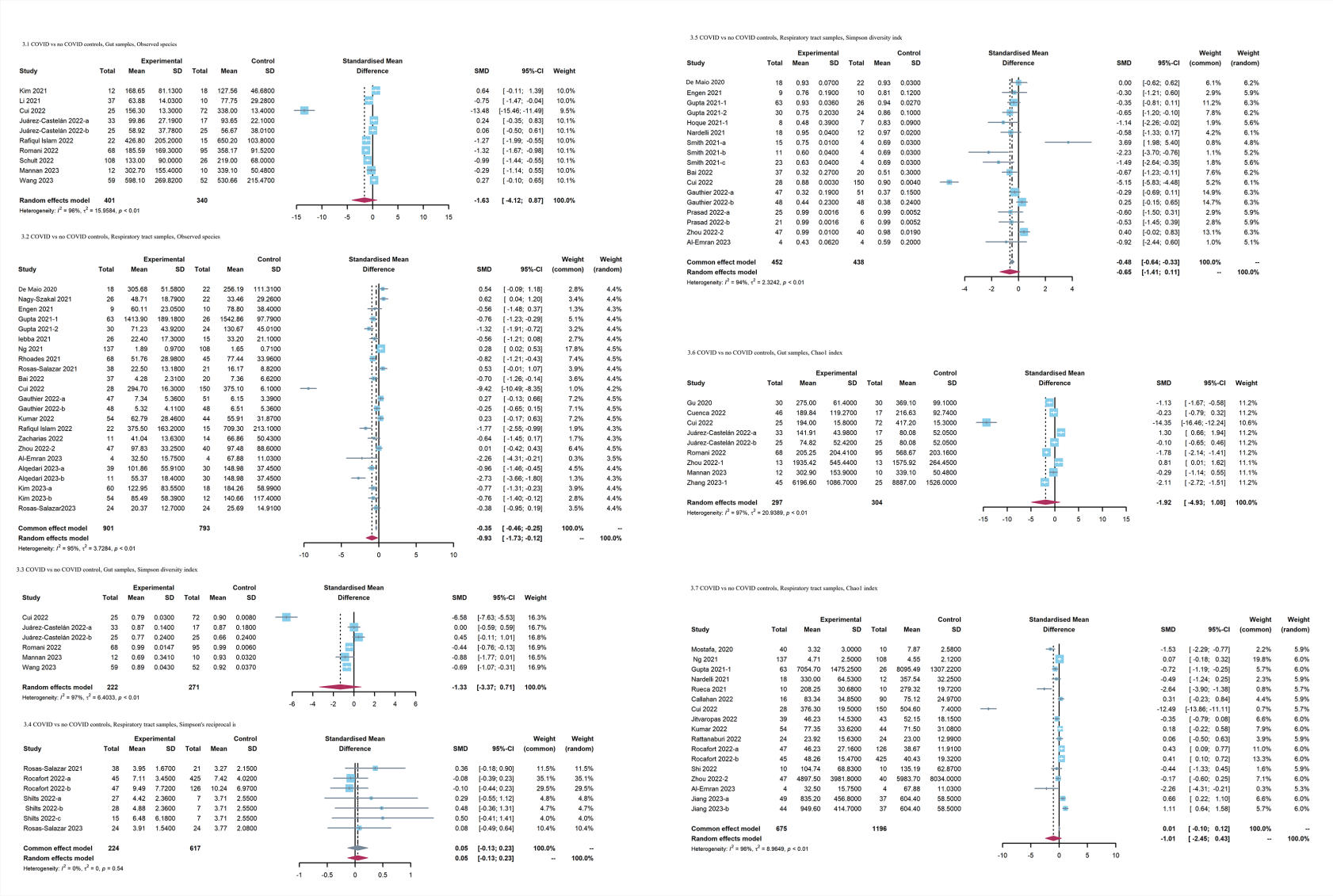
Supplementary Figure 1 Forest plots of α-diversity in the gut and respiratory tract microbiota of patients with COVID-19 compared with no COVID-19 controls.SD, standard deviation; CI, confidence interval.


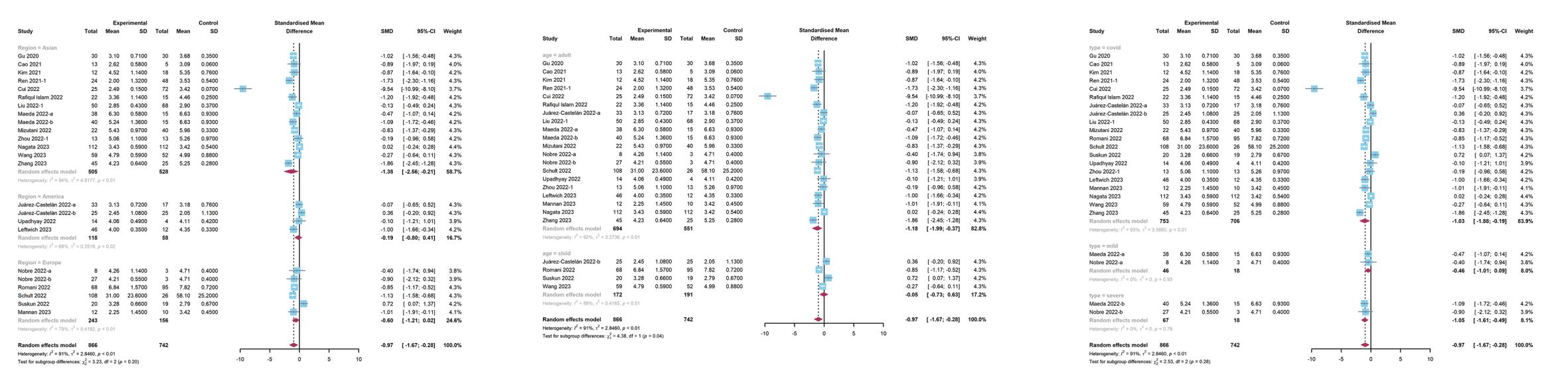


Supplementary Figure 2 Subgroup analysis results of Shannon index in the gut of COVID-19 patients versus no COVID-19 control. SD,standard deviation; CI, confidence interval.


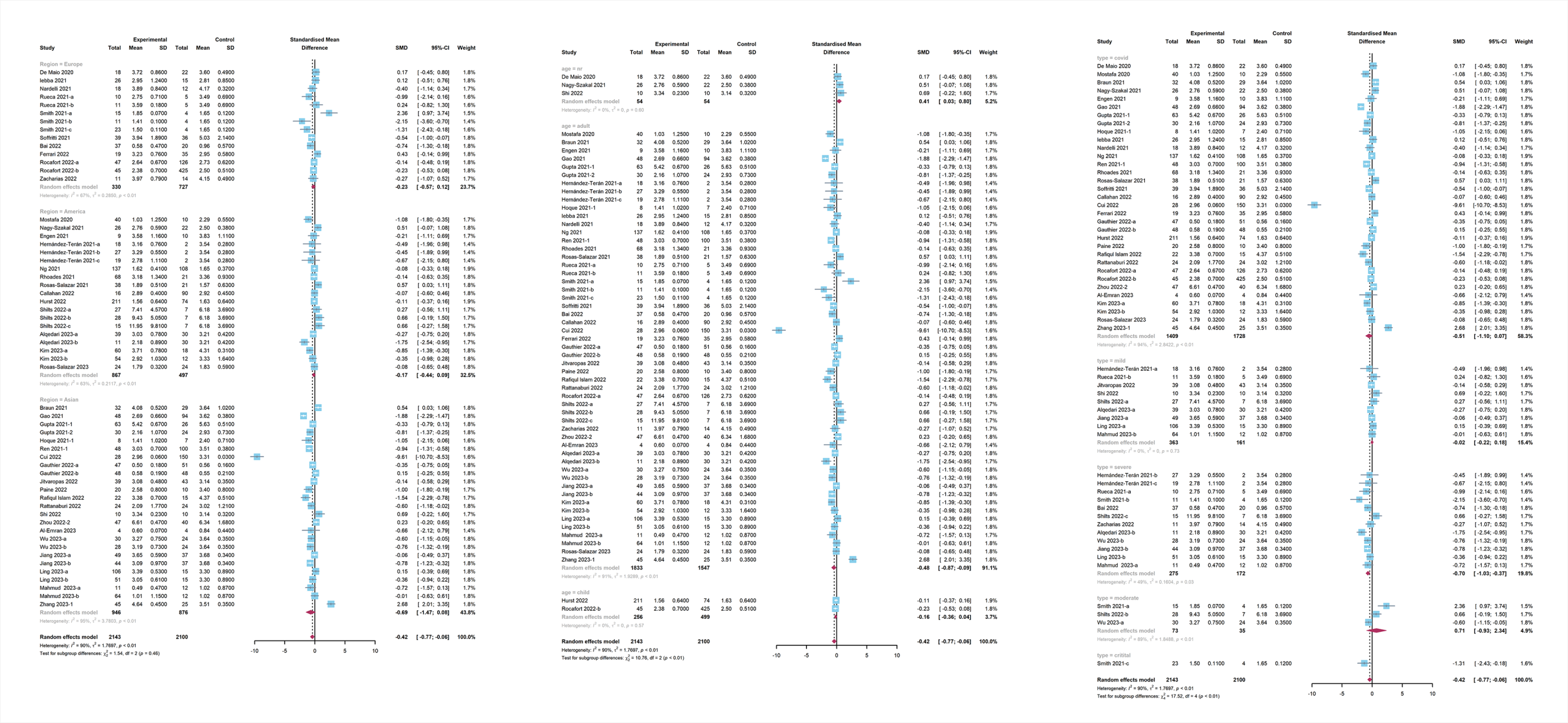
Supplementary Figure3 Subgroup analysis results of Shannon index in the respiratory tract of COVID-19 patients versus no COVID-19 control. SD, standard deviation; CI, confidence interval.
